# Mental health practitioners’ perceptions and adoption intentions of AI-enabled technologies: an international mixed-methods study

**DOI:** 10.1186/s12913-025-12715-8

**Published:** 2025-04-16

**Authors:** Julia Cecil, Anne-Kathrin Kleine, Eva Lermer, Susanne Gaube

**Affiliations:** 1 Department of Psychology, LMU Center for Leadership and People Management, LMU Munich, Geschwister-Scholl-Platz 1, Munich, 80539 Germany; 2 Department of Business Psychology, Technical University of Applied Sciences Augsburg, An der Hochschule 1, Augsburg, 86161 Germany; 3 UCL Global Business School for Health, University College London, 7 Sidings St, London, E20 2 AE UK

**Keywords:** Mental healthcare, Artificial intelligence, Technology implementation, Use intention, Learning intention

## Abstract

**Background:**

As mental health disorders continue to surge, exceeding the capacity of available therapeutic resources, the emergence of technologies enabled by artificial intelligence (AI) offers promising solutions for supporting and delivering patient care. However, there is limited research on mental health practitioners’ understanding, familiarity, and adoption intentions regarding these AI technologies. We, therefore, examined to what extent practitioners’ characteristics are associated with their learning and use intentions of AI technologies in four application domains (diagnostics, treatment, feedback, and practice management). These characteristics include medical AI readiness with its subdimensions, AI anxiety with its subdimensions, technology self-efficacy, affinity for technology interaction, and professional identification.

**Methods:**

Mixed-methods data from *N* = 392 German and US practitioners, encompassing psychotherapists (in training), psychiatrists, and clinical psychologists, was analyzed. A deductive thematic approach was employed to evaluate mental health practitioners’ understanding and familiarity with AI technologies. Additionally, structural equation modeling (SEM) was used to examine the relationship between practitioners’ characteristics and their adoption intentions for different technologies.

**Results:**

Qualitative analysis unveiled a substantial gap in familiarity with AI applications in mental healthcare among practitioners. While some practitioner characteristics were only associated with specific AI application areas (e.g., cognitive readiness with learning intentions for feedback tools), we found that learning intention, ethical knowledge, and affinity for technology interaction were relevant across all four application areas, underscoring their relevance in the adoption of AI technologies in mental healthcare.

**Conclusion:**

In conclusion, this pre-registered study underscores the importance of recognizing the interplay between diverse factors for training opportunities and consequently, a streamlined implementation of AI-enabled technologies in mental healthcare.

**Supplementary Information:**

The online version contains supplementary material available at 10.1186/s12913-025-12715-8.

## Introduction

One in eight people worldwide is affected by a mental disorder, and the trend is rising [1]. Frequently, the demand for therapeutic support exceeds available resources, especially since the number of mental health practitioners is not increasing quickly enough [2]. Simultaneously, technologies enabled by artificial intelligence (AI) are advancing and gaining relevance in the support and delivery of patient care, owing to their potential for improving patient outcomes through an early detection of mental disorders and personalized treatment [3], and facilitating the work of practitioners [4]. Given the proposed benefits, AI-enabled technologies provide an opportunity to bridge the gap between mental healthcare needs and available therapeutic resources.

### Applications of AI-enabled technologies in mental healthcare

AI-enabled technologies refer to systems or applications characterized by humanlike capabilities, including decision-making through problem solving and continuous learning [3]. To execute their tasks effectively, these technologies rely on large amounts of data. Common data sources for AI-enabled technologies in mental healthcare include behavioral data (e.g., video and audio recordings), followed by biological (e.g., blood samples) and neuroimaging data (e.g., electroencephalogram) [5]. Within mental healthcare, we suggest that AI-enabled technologies utilized by clinicians that leverage these datasets can be broadly categorized into four application areas: diagnostic support, treatment support, feedback, and practice management.

The first two application areas, diagnostic and treatment support, refer to patient-centered technologies. *Diagnostic* applications leverage AI to enhance the accuracy and efficiency of mental health assessments by evaluating a range of patient data, such as genetic information, language, voice, and facial expressions [6–8]. For example, certain tools can distinguish between diagnoses that share similar symptoms but require different treatment approaches, such as various types of dementia or bipolar and unipolar depression [9].

The second area of technologies provides *treatment* support, making mental health treatments more personalized and precise [10]. These technologies are predominantly working with genetic, neuroimaging, clinical and demographical datasets [11]. For instance, AI-enabled technologies can be utilized at the beginning of therapy to estimate a patient’s potential response to different medications, such as antidepressants, or to predict remission rates [11].

Besides these patient-centered technologies, an increasing number of practitioner-centered applications are emerging, with the third area comprising *feedback* tools for mental health professionals: These types of applications aim to provide practitioners with feedback on the quality of their patient interactions by evaluating session data, for instance, through speech signals and the language patterns of the interaction [12–15]. Feedback reports usually include an assessment of the session’s strengths and potential areas for improvement, such as increasing the times for reflections or including more open-ended questions [16].

Finally, the fourth application area of AI-enabled technologies for mental health is *practice management.* They are supposed to automate clinical and administrative workflows and thereby reduce the administrative burden for mental healthcare professionals [16]. For example, by automatically transcribing therapy sessions using speech data and integrating the transcripts into medical records [16], patient data entry can become more efficient and structured [17].

### Adoption of AI-enabled tools in mental healthcare and its antecedents

The proposed benefits of using AI tools such as an early detection of mental disorders, increasing patient access, and personalized treatment will only be realized if practitioners use them as intended [7]. However, studies show widespread skepticism regarding the use of AI-enabled technologies in healthcare [4, 9, 18–20]. A lack of understanding or knowledge of the mechanisms and processes underlying the technology may explain some of the suspicion that impacts the uptake of technologies [10, 21]. For instance, limited working knowledge of machine learning algorithms increases the risk of misinterpretation and misuse [10], while their opaque and complex nature can reinforce resistance among mental health practitioners [21]. Therefore, gaining deeper insights into the current state of mental health practitioners’ understanding of and experiences with AI-enabled tools is the first step to recognize barriers to the adoption and determine starting points for measures aimed at promoting safe technology practices. However, to the best of our knowledge, no study has investigated practitioners’ understanding of AI-enabled tools for mental healthcare (RQ1), their familiarity with these technologies (RQ2), in what context they learned about them (RQ3), and whether they have used any of these tools in their clinical practice (RQ4). Besides knowledge and exposure, technology acceptance and effective use is influenced by numerous individual variables.

### The role of learning in the adoption of AI-enabled technologies

**Fig. 1:**
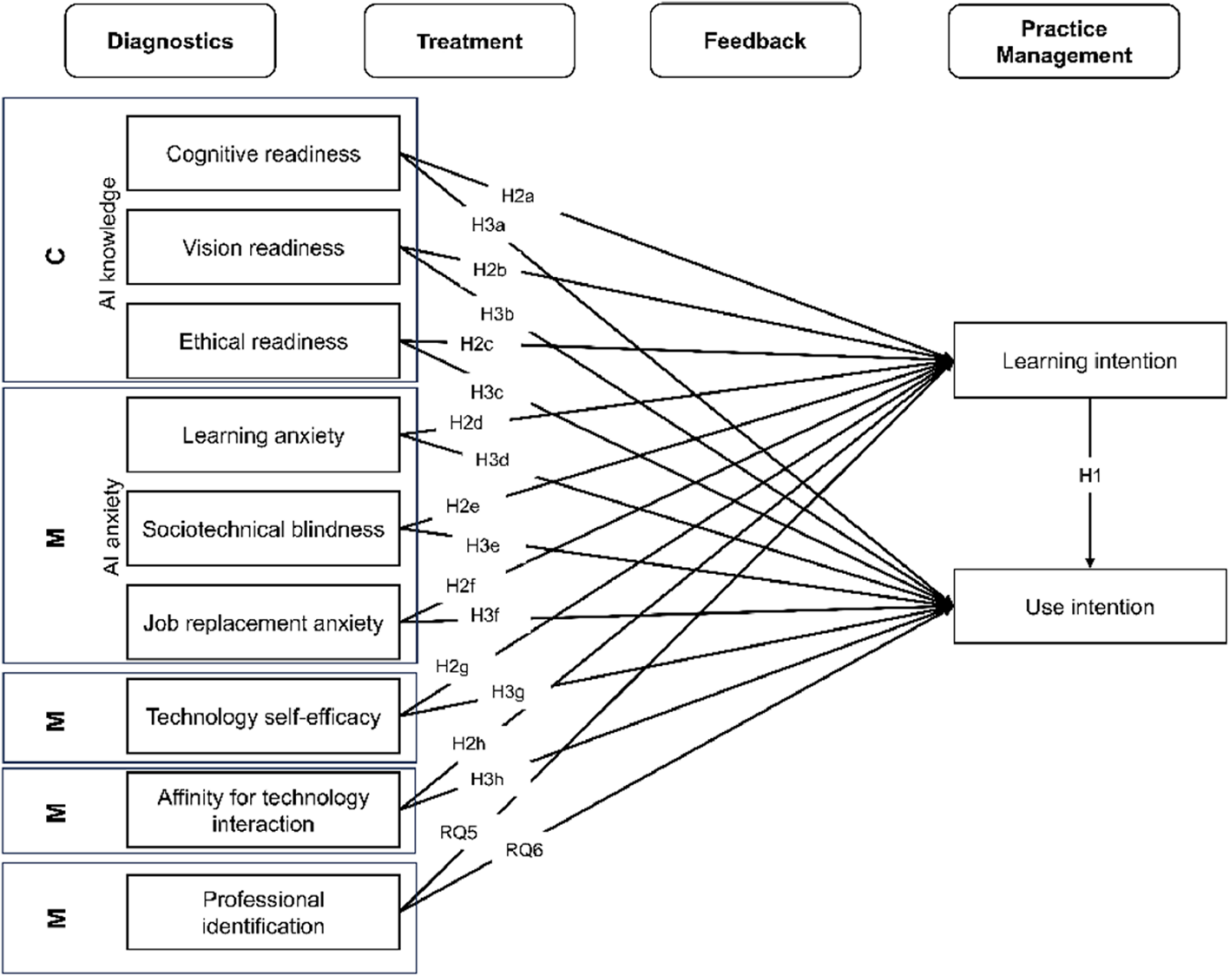
Proposed research model for each of the following application areas: diagnostics, treatment, feedback, and practice management. Components of the COM-B model [30] are abbreviated as followed: C = Capability, M = Motivation

Studies have highlighted the pivotal role of learning opportunities and training in the implementation process by equipping healthcare professionals with the requisite skills to effectively use AI-enabled technologies in their practice [22–24]. Conversely, healthcare professionals ranked the lack of instruction and training on technology use as the primary technology-related cause of medical errors [25]. Training is believed to reduce the perceived risk associated with using such tools and, further, minimize the workload arising from the implementation of AI technologies [26]. It has been shown that the willingness to receive training about an AI technology is positively associated with clinicians’ use of it, as training can help reduce AI-related workload and alleviate concerns about AI-associated risks [26]. We, therefore, hypothesized that *learning intention* is positively associated with *use intention* for AI-enabled technologies in mental healthcare (H1). Figure 1 depicts the proposed model with the related hypotheses and research questions. However, learning intentions and use intentions represent different levels of engagement with technologies. The willingness to learn and receive training is a rather theoretical interaction with a technology centered around updating knowledge [27]. Yet, use intention implies the willingness to make the necessary effort to use the technology in practice [28, 29]. Hence, it is important to study both the learning and use intention and their respective antecedents independently.

### Individual-level factors in the adoption of AI-enabled technologies

Most studies have focused on AI adoption in general healthcare settings (see [31] for a review) or different medical specialties such as dermatology [32]. However, less is known about individual-level factors associated with practitioners’ intentions to learn about and use AI-enabled technologies in mental healthcare. User characteristics represent one of the key determinants for the adoption of healthcare technologies [33]. Research showed that common demographic and individual differences such as gender [34], age [35], personality [31, 32, 36], and country of residence [37, 38] influence technology uptake. Further, practitioners’ intention to use AI-enabled technologies in mental health is greatly influenced by their individual beliefs, attitudes, and perceptions [18]. Hence, this study seeks to extend existing literature by systematically investigating individual factors that contribute to a holistic understanding of the determinants affecting the learning and use intention of AI-enabled technology in mental healthcare. While technology acceptance theories, such as the Technology Acceptance Model (TAM [30]) and the Unified Theory of Acceptance and Use of Technology (UTAUT [29]) have been employed to explain AI adoption (see [39]), the Capability-Opportunity-Motivation Behavior (COM-B) model developed by Michie et al. [40] offers a complementary perspective. As a well-validated behavior change theory, COM-B has been successfully used in synthesizing and understanding healthcare-related technology adoption (for instance, see [41, 42]). The COM-B model indicates that individuals’ capabilities, motivation, and opportunities determine their behavior [40]. Capability is defined as an individual’s psychological and physical ability required for a particular behavior, including the essential knowledge and skills. Motivation encompasses reflective or automatic cognitive processes that direct behavior, extending beyond conscious decision-making to habitual patterns, emotional responses, and analytical reasoning. Opportunity relates to external factors lying outside an individual’s immediate control that influence behavior, including social and physical opportunity [40]. Upon reviewing the empirical literature, we identified the most important individual-level factors relevant to technology adoption and ultimately integrated them into the COM-B framework. As opportunity includes factors outside the individual, we focused on the domains of capabilities and motivations.

First, individuals’ capability is important for engaging in a respective behavior [40]. Different aspects of capability, including AI knowledge, have been found to be relevant for AI adoption. A positive relation between AI knowledge and the intention to use AI technology was found among prospective physicians [43] and among prospective therapists for feedback providing AI tools [20]. Similarly, a lack of technology-related skills and knowledge among therapists was identified as a barrier in the use of technology in forensic psychiatry [44]. However, one study found no significant association between AI knowledge and medical students’ intention to learn about AI [45]. As AI knowledge referred to different aspects in each study, and the mixed findings consequently might have resulted from methodological differences, we are adopting a broader construct called *readiness for medical AI*. Readiness for medical AI can be divided into different subdimensions [46]: *Cognitive readiness* encompasses peoples’ cognitive abilities such as knowledge of and critical thinking about AI technologies. *Vision readiness* involves the ability to envision and anticipate the potential impact, benefits, and challenges associated with AI technologies. *Ethical readiness* refers to an individual’s awareness, knowledge and adherence to ethical standards or guidelines for the use of AI technologies. The relationship between the subdimensions of medical AI readiness and the learning and use intentions of AI-enabled technologies in mental healthcare has not been examined in-depth. Only one study found a positive association between cognitive readiness and the intention to use a feedback tool in mental healthcare [20]. We expected that cognitive readiness (H2a, H3a), vision readiness (H2b, H3b), and ethical readiness (H2c, H3c) are all positively associated with the learning and use intentions of AI tools for mental health (see Fig. 1 for all hypotheses).

Second, automatic motivational processes influence a particular behavior [40]. In the context of technology adoption, automatic processes like emotions, as a sub-component of motivation, have been shown to have an influence [40]. Usually, negative valanced variables, such as *AI anxiety*, have been investigated [47]. *AI anxiety* refers to the apprehension, concern, or fear experienced in response to the implementation, use, or potential consequences of AI technologies [48]. The construct encompasses three subdimensions: *learning anxiety*, *sociotechnical blindness*, and *job replacement anxiety* [47]. *Learning anxiety* refers to the anxiety regarding acquiring knowledge and skills related to AI technologies. S*ociotechnical blindness* relates to anxiety arising from a lack of understanding that AI systems currently do not operate independently without human oversight. *Job replacement anxiety* refers to a person’s fear that their occupation will be replaced or disrupted by AI technologies [36, 49]. Y.-M. Wang et al., showed that AI learning anxiety negatively affected intrinsic and extrinsic learning motivation [47]. They also found that job replacement anxiety positively influenced extrinsic but not intrinsic learning motivation, indicating that some people might only gain AI-relevant skills and knowledge to avoid unemployment. Regarding use intentions, technology anxiety emerged as one important barrier of technology use in healthcare [50]. AI anxiety correlated negatively with the use intention of AI-based technology in healthcare among nurses [51] and the intention to use AI-based treatment and feedback tools among prospective psychotherapists [20]. While there is consistent evidence, that AI anxiety hinders AI adoption, none of these studies explored associations between all three subdimensions and learning and use intentions for AI-enabled technologies simultaneously. Therefore, we incorporated all three subdimension separately into our research model. We hypothesized that AI learning anxiety (H2d, H3d) and sociotechnical blindness (H2e, H3e) are negatively associated with both the learning and use intentions of AI tools. Job replacement anxiety is thought to be positively associated with the AI learning intentions (H2f) and negative with use intentions (H3f).

Third, in addition to automatic motivational processes, reflective processes, are also crucial, with self-efficacy being an important factor influencing behavior uptake [40]. The subcategory tailored to technology is *technology self-efficacy* which refers to a person’s belief in their capacity to effectively accomplish a technologically advanced task [52]. It is well established that *technology self-efficacy* is an important predictor of technology adoption in healthcare [53]. Higher technology self-efficacy has been positively associated with medical students’ intention to learn technologies [45], healthcare professionals’ readiness to adopt technologies [54] as well as their intention to use nursing apps and AI technology [51, 55, 56]. In accordance with this large body of research, it is hypothesized, that technology self-efficacy is positively associated with AI learning and use intentions among mental health practitioners (H2g, H3g).

Fourth, *affinity for technology interaction* represents another motivational process. It serves as a fundamental resource for technology adoption as it is characterized as the tendency to proactively partake in extensive technological interaction [57]. Higher affinity for technology was positively related to using a wider range of learning strategies for different healthcare systems among physician trainees [58]. Among clinicians, a positive association between affinity for technology and attitude towards technology use has been found and higher technology affinity was linked to a preference for more advanced technologies [59, 60]. To the best of our knowledge, the relationship between affinity for technology interaction and the intention to learn or use AI technologies in mental healthcare has not been investigated. Based on previous evidence from the medical context, we hypothesized that affinity for technology interaction is positively associated with AI learning and use intentions (H2h, H3h).

Finally, the relevance of people’s perception of their social and professional role and identity as a motivational factor has also been highlighted in the context of technology adoption, often through *professional identification.* Professional identification refers to the degree to which an individual feels a deep connection and unity with their chosen occupation [61]. Professional identification plays an important role in the adoption of novel work behavior [61], particularly important with the integration of AI-enabled technologies that affects practitioners’ daily tasks [62]. However, changes in the workplace are likely to be resisted if they are perceived as a threat to professional identity [63]. It has been shown that threats to professional identity directly impacted healthcare practitioners’ technology use [64]. Moreover, aligned professional beliefs with the designated roles of technology are fundamental for technology adoption [65] as one’s professional identification influences technology integration [63]. Given these insights, the following research questions are proposed as we could not derive a clear direction of the effects from the literature: Is professional identification associated with AI learning intention (RQ5) and AI use intention (RQ6)?

Prior research has shown that there are differences in use intentions and its predictors across AI tools for different application areas [20]. As AI-enabled technologies in mental healthcare differ vastly in their purpose, they might also be perceived differently by mental health practitioners. Therefore, we believe it is important to look at the learning and use intentions and their antecedents individually for each application area. Providing such a nuanced understanding enables technology developers and healthcare organizations who purchase these technologies to consider the factors relevant to the tool in question, thereby facilitating a more efficient and safe design and implementation process. As a consistent methodology that allows comparisons across the different application areas on the same level is fundamental for this, we applied the same research design and sample across all four application areas of AI-enabled technologies in mental healthcare. This allows us to systematically identify potential differences, ultimately resulting in a comprehensive overview of different application areas and their antecedents.

### The present study

The main goal of this mixed method study was twofold. First, we want to investigate mental health practitioners’ general understanding, familiarity, and experience with AI technologies (RQ1 – RQ4) and their attitudes towards different application areas of AI-enabled tools using qualitative content and descriptive analysis. In this line, we also examined differences in attitudes toward technology across different professions, gender, and countries. Second, this work aims to provide a differentiated insight into factors associated with learning and use intentions of AI-enabled technologies for mental health, separated by application areas (H1, H2a – H2h, H3a – 3h, and RQ5 and RQ6). Gaining a deeper understanding of the relative importance of individual factors might help for deriving training and intervention strategies tailored specifically towards practitioners’ needs for different technology application areas.

## Methods

### Participants

Data for the pre-registered (https://osf.io/9jxwy/) cross-sectional, mixed-methods survey study was collected between July and October 2023. Participants included psychotherapists in training, psychotherapists, psychiatrists, and clinical psychologists. Participants were recruited via emails distributed among universities and psychotherapy training institutes in Germany and the US, social media postings, and Prolific. The online survey was available in German and English language. For the German version of the survey, all items were translated using back-and-forth translation. The English version of the survey can be found in the online Supplementary Material 1 and the German version on OSF (https://osf.io/9jxwy/). In total, 670 mental health practitioners agreed to participate, of which 227 did not finish the survey and 51 failed at least one attention check item, resulting in *N* = 392 participants included in the data analysis. This number exceeds the average response rates in surveys [66] and the minimum sample size determined by the a priori power analysis for structural equation modeling (SEM), which required at least 50 practitioners per country (Germany and US). Demographic information of the included participants can be found in Table 1. The study was approved by the Ethics Committee of the University of Regensburg (23–3365- 101).

**Table 1:** Participant demographics

	N (%) or M (SD)
Age	34.34 (10.46)
Gender	
Female	291 (74.2%)
Male	92 (23.5%)
Non-binary/third gender	7 (1.8%)
NA	2 (0.5%)
Profession^a^	
Psychotherapist in training	235 (60.0%)
Psychotherapist	73 (18.6%)
Psychiatrist	39 (9.9%)
Clinical psychologist	42 (10.7%)
Others	3 (0.8%)
Therapeutic approach^a^	
(Cognitive) behavioral therapy	276 (56.8%)
Psychodynamic therapy	94 (19.3%)
Psychoanalytic therapy	29 (6.0%)
Systemic therapy	30 (6.2%)
Other^b^	57 (11.7%)
Workplace	
Practice	69 (13.5%)
Private practice	63 (12.3%)
General hospital (i.e., Psycho-oncology)	30 (5.9%)
Specialist hospital for psychiatry, psychotherapy, psychosomatic medicine or neurology	118 (23.0%)
Rehabilitation clinic	26 (5.1%)
(University) outpatient clinic	112 (21.9%)
Community mental health center/counseling center	48 (9.4%)
Other	46 (9.0%)
Professional experience (in years)	5.89 (7.26)

*N* = 392, n_German_ = 236; n_US_ = 156; NA = participants preferred not to answer

^a^ Multiple answers possible

^b^ the list with the final data can be found on OSF

### Procedure

First, demographic and occupation-related information was assessed in the survey. Second, participants’ understanding of, familiarity and experiences with, and use of AI-enabled tools were assessed. Third, participants were then introduced to the four different application areas of AI-enabled technologies in mental health. For each area, participants received a short description and an example (see Table 2), derived from existing research and applications (see online Supplementary Material 2). We measured learning and use intentions as dependent variables for each application area, the individual level factors as predictor variables, several control and occupation-related variables (occupation, therapeutic approach, workplace, working experience in years) as described in the subsequent section.

**Table 2:** Description of the different application areas shown to the participants

Application area	Description	Example
Diagnostics	AI-enabled methods are used to screen or diagnose mental disorders. This can be done, for example, by analyzing the patient’s speech, voice, facial expressions, or other patient data.	*Speech software for a more differentiated determination of the severity of the patient’s depression.*
Intervention and treatment	AI-enabled methods are used to support interventions and treatments and/or enable (personalized) therapy and/or intervention recommendations.	*Algorithmic analysis of biological markers for the selection of psychotropic drugs individually tailored to the patient.*
Feedback for practitioners	AI-enabled methods are used to provide practitioners with feedback on their therapeutic work (especially conversational skills).	*Software that analyzes audio recordings of therapy sessions and produces a report on strengths (e.g.*,* optimal use of reflections) and suggestions for improvement (e.g.*,* more open questions).*
Practice management/organization	AI-enabled methods are used to automate administrative tasks and practice management.	*Automated processing of inquiries (e.g.*,* frequently asked questions*,* appointments) or automated integration of audio recordings of sessions into medical records.*

### Measurements

#### Understanding

Participants were asked to describe what they understand by AI-enabled technologies in the field of psychotherapy/psychiatry and how they could be used in their daily work in their own words, using an open text box.

#### Familiarity

Next, they were asked to choose one of three options regarding their familiarity with AI-enabled technologies (a: “I have never heard of AI-enabled technologies in psychotherapy/psychiatry”; b: “I have heard of AI-enabled technologies in psychotherapy/psychiatry”; c: “I have actively looked into AI-enabled technologies in psychotherapy/psychiatry”). Participants who had stated to have heard of AI-enabled technologies were asked in which context they did so (open question). Participants who had stated that they actively looked into AI technology, were given three context options: “I have informed myself independently (e.g., online,…)”, “I attended voluntary information sessions on AI-enabled technologies in psychotherapy/psychiatry”, and “I have participated in trainings on this topic (e.g., to get training points).”

#### Use

To determine previous use, participants were asked to state whether they had used AI-enabled technologies in their clinical practice (yes/no).

### Dependent variables

Two dependent variables, *learning intention* and *use intention*, were assessed for each of the four described application areas for AI tools in mental health. Learning intention was measured with “I intend to learn about AI technologies in [application area]” on a 5-point Likert scale from 1 (*strongly disagree*) to 5 (*strongly agree*) based on Venkatesh et al. [29]. Similarly, use intention was assessed with the item “I intend to use AI technologies in [application area] in my work” with the same response format [29].

### Predictor variables

#### Medical AI readiness

*Cognitive*,* vision* and *ethical readiness for medical AI* was based on the Medical Artificial Intelligence Readiness Scale (MAIRS) from Karaca et al. [46]. For each of the subscales we omitted items for two reasons. First, items measuring the actual use of technology were removed, as we assumed that most practitioners are not currently using AI-enabled tools and therefore these questions could not be answered properly. Second, items with low factor loadings were removed to keep the survey reasonably short. Consequently, we included 11 items, rated on a 5-point Likert scale from 1 (*strongly disagree*) to 5 (*strongly agree*). The scale showed acceptable (𝛼_Vision_ = 0.79, 𝛼_Ethics_ = 0.73) to good internal consistency (𝛼_Cognition_ = 0.81).

#### Anxiety

*AI learning anxiety*,* job replacement anxiety* and *sociotechnical blindness* were assessed using the 18-item Artificial Intelligence Anxiety Scale (AIAS) by Wang & Wang [49] on a 7-point Likert scale from 1 (*strongly disagree*) to 7 (*strongly agree*). The internal consistency of the sociotechnical blindness subscale was acceptable (𝛼_Sociotechnical_ = 0.78), that of the job replacement anxiety subscale good (𝛼_Jobreplacement_ = 0.87) and that of the AI learning anxiety subscale was excellent (𝛼_Learning_ = 0.93). 

#### Affinity for technology interaction 

*Affinity for technology interaction* was measured with the Affinity for Technology Interaction Scale (ATI-S [67]). The four items were rated on a 7-point Likert scale from 1 (*completely disagree*) to 7 (*completely agree*). The scale showed good internal consistency (𝛼_Affinity for technology_ = 0.81). 

#### Technology self-efficacy

*Technology self-efficacy* was assessed using the five-item scale of McDonald and Siegall [52] on a 7-point Likert scale from 1 (*strongly disagree*) to 7 (*strongly agree*). The internal consistency of the scale was acceptable (𝛼_Technology self−efficacy_ = 0.71).

#### Professional identification

*Professional identification* was measured using the five items from Hekman et al. [61] on a 5-point Likert scale from 1 (*strongly disagree*) to 5 (*strongly agree*). The scale showed acceptable internal consistency (𝛼_Professional identification_ = 0.77).

### Control variables

Age, gender, and personality were included as control variables based on research showing that all three variables have an impact on technology adoption [31, 32, 34–36]. Participants‘ personality traits were assessed using the Big Five Inventory [68], on a 5-point Likert scale from 1 (*strongly disagree*) to 5 (*strongly agree*), including the main dimensions openness, conscientiousness, extraversion, agreeableness, and neuroticism. The internal consistency of four of the Big Five subscales ranged from to acceptable to good (𝛼_Openness_ = 0.72; 𝛼_Conscientiousness_ = 0.77; 𝛼_Extraversion_ = 0.84; 𝛼_Neuroticism_ = 0.74), with only the subscale agreeableness showing a sufficient internal consistency (𝛼_Agreeableness_ = 0.58) [68].

### Data analysis

Data was analyzed using R (Version 4.3.2, R Core Team, 2023). Answers to the open questions were coded using Excel.

### Qualitative and descriptive analysis

First, we conducted a qualitative content analysis to get in-depth insights into mental healthcare practitioners’ understanding of AI-technology for their field of work (RQ1), and allowing for participants’ viewpoints to emerge [69]. To gain these insights, we used a deductive thematic analysis [70] to identify how many types of AI applications were mentioned by practitioners. Participants’ responses were clustered into the four predefined application areas and then analyzed for their frequency, to gain insights about the most known and common areas. Further, the precision of their description of AI-enabled technologies in mental healthcare was assessed. We examined whether practitioners could not give a description if the descriptions solely included the technology’s potential area of application or if also the tool’s underlying functions or operational mechanism were explained properly. For answers to the open question regarding the context in which they have heard about the AI technologies (RQ3), an inductive approach [70] was employed to identify recurrent categories within the data. Participants’ responses were coded based on similarities and organized subsequently into themes representing higher-level concepts. All responses were independently coded by two researchers to review and validate the identified themes with subsequent discussion in cases with coding discrepancies. The code book can be found in the online material on OSF (https://osf.io/9jxwy/).

### SEM

Next, to look at the learning and use intentions, we specified one SEM model for each application area using the ‘lavaan’ package [71]. Confirmatory factor analyses (CFA) were calculated for each model. For the model fit, root-mean-square error of approximation (RMSEA) values smaller than 0.05 are considered good and smaller than 0.08 acceptable [72]. Standardized root-mean-square residual (SRMR) values up to 0.08 are considered satisfactory [73]. Models showing comparative fit index (CFI) and Tucker Lewis index (TLI) values near to or surpassing 0.90 possess a reasonable level of fit [73]. For each application area, we analyzed models to predict learning and use intention from the predictor variables and the control variables age, gender, and personality. Further, we calculated three more parsimonious theoretical models to avoid overfitting and ensure the distinctness of the variables. For the first parsimonious model, we combined the subscales of readiness for medical AI. In the second parsimonious model, the subscales of AI anxiety were merged, and in the third parsimonious model, affinity for technology interaction and technology self-efficacy were combined. All in all, SEMs were calculated for one research model per application area with and without control variables, as well as the three more parsimonious models, totaling eleven models.

### Explorative analysis of demographic and tool differences

Finally, for the analysis of potential group differences, we assessed the mean values, standards deviations, and correlations between the variables used in the SEM. Group differences across the four application areas and practitioners’ subgroups (profession, gender, country) were assessed using t-tests or one-way ANOVAs with post-hoc Tukey-HSD. The data was found to be normally distributed following testing for assumptions, with only minor violations observed for learning and use intentions. However, simulation studies demonstrated that, particularly in studies with larger samples, such violations have a negligible impact on the results [74]. Additionally, familiarity and use experiences with AI-enabled technologies among mental health practitioners and their context (RQ2 - 4) were analyzed descriptively.

## Results

### Practitioners' understanding and familiarity with different application areas

When participants were asked to explain their understanding of AI-enabled technologies in mental healthcare and how they could be used in their daily work in their own words, 10.5% could not provide a description. Over half of those that provided a description (53.7%) mentioned only one application area, while a further 37.6% stated two categories (RQ1). Merely 8.1% of participants named three areas, whilst only 0.6% of participants (*n* = 2) listed all four. AI-enabled tools for supporting treatment decisions emerged as the most frequently mentioned area (69.8%), followed by diagnostic (43.4%) and practice management tools (41.1%). Only six participants mentioned feedback tools (1.7%). Participants exhibited varying levels of precision in the description of these technologies, however mostly demonstrating a basic understanding through their explanations. While a majority provided less detailed statements, such as indicating AI’s role as “diagnostic assistance” (Clinical psychologist, 45), a minority offered more elaborate descriptions, exemplified by one professional’s description that “AI could help to make diagnosis […] more efficient and precise by pooling larger data sources together (e.g., interview data, EHR data, patient-reported outcomes, biomarker data)” (Clinical psychologist, 47). For treatment tools, most participants also solely addressed their general purpose, such as “tools that have been programmed to respond to folks in crisis” (Psychotherapist in training, 32). A smaller subset displayed a deeper understanding by mentioning the underlying working mechanism: “By considering an individual’s unique history, symptoms, and responses to therapy, AI can recommend specific interventions and strategies tailored to their needs” (Psychiatrist, 69). Professionals mostly described feedback tools briefly as tools that “give input into your performance as a therapist” (Clinical psychologist, 26). Only two participants provided additional information by stating that “there are programs that listen to and transcribe therapy sessions and from this identify themes, relational patterns, and can even rate the therapist on various qualities and suggest interventions” (Clinical psychologist, 35). Likewise, a disparity in the precision level of participants’ responses emerged about practice management tools, ranging from succinct descriptions, such as “documentation of visit” (Psychiatrist, 46) and “can be used to write notes” (Psychotherapist, 34) to more elaborate insights: “I think predictive text could be used for things like notes and that AI software can be used for recording and transcribing sessions, and then generating notes” (Clinical psychologist, 33).

### Experiences of mental health practitioners with AI-enabled technologies

**Fig. 2:**
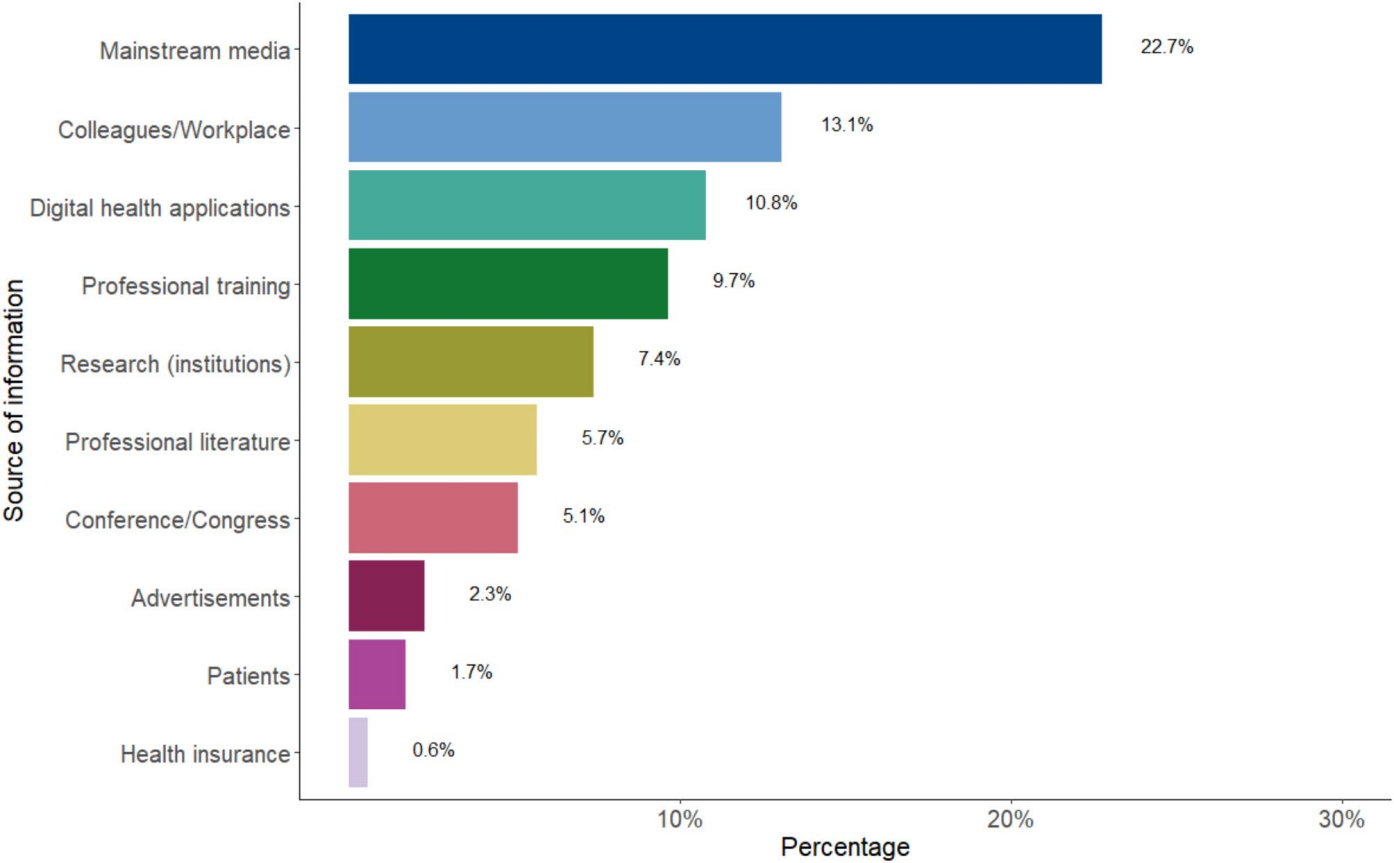
Distribution of sources of information regarding AI-enabled technology for mental health. Responses from participants who heard of AI in mental health (*n* = 176). Mainstream media included media coverage, news, internet, social media, podcasts, and newspaper articles

Nearly half of the practitioners (*n* = 178, 45.4%) stated that they have never heard of AI-enabled technologies in the field of psychotherapy/psychiatry, while 44.9% (*n* = 176) did (RQ2). Figure 2 displays their sources of information. Overall, only 9.7% (*n* = 38) actively looked into this topic, whose majority obtained information independently through online research (*n* = 29, 76.3%). A further 10.5% (*n* = 4) stated that they attended voluntary information sessions and only 13.2% (*n* = 5) participated in formal trainings (RQ3). The vast majority of participating practitioners (*n* = 366, 93.37%) have not used AI-enabled technologies in their clinical practice (RQ4).

### Learning and use intentions across application areas

**Fig. 3:**
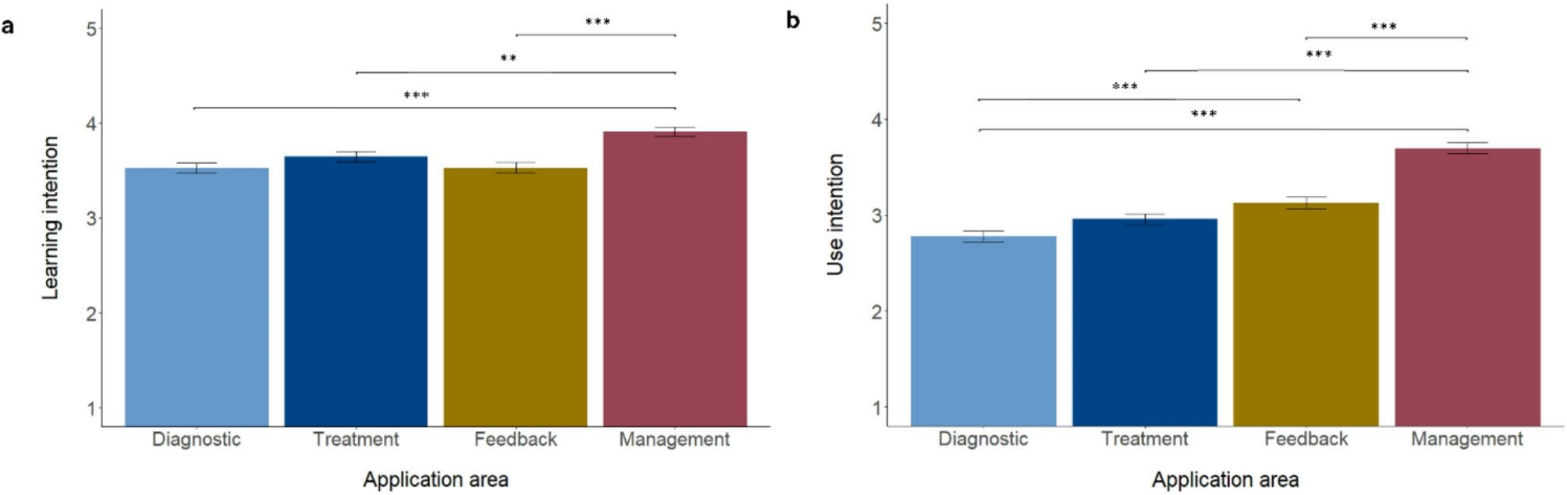
**a** Learning intentions and **b** use intentions across the different application areas. * *p* ≤ 0.05; ** *p* ≤ 0.01, *** ≤ 0.001

The data were normally distributed, with mild violations for learning and use intentions. However, simulation studies showed that especially for larger samples as in our study, mild violations have little to no effect on the results. The overall learning intention was significantly higher than the overall use intention, *t*(781) = 8.17, *p* < 0.001, d = 0.584; *M*_Learning_ = 3.65, *SD*_Learning_ = 0.88; *M*_Use_ = 3.14, *SD*_Use_ = 0.88). Further, both differed across the four application areas. Practitioners’ intention to learn was significantly higher for AI-enabled management tools (*M* = 3.91, *SD* = 1.01) compared to diagnostic (*M* = 3.53, *SD* = 1.12), treatment (*M* = 3.65, *SD* = 1.09), and feedback tools (*M* = 3.53, *SD* = 1.19; *F*(3, 1564) = 10.38, *p* < 0.001, η_p_^2^ = 0.02; see Fig. 3a). Practitioners’ use intentions were significantly higher for AI-enabled tools for feedback (*M* = 3.13, *SD* = 1.22) than diagnosis (*M* = 2.78, *SD* = 1.15) and again, for management tools (*M* = 3.70, *SD* = 1.10) compared to diagnosis, treatment (*M* = 2.96, *SD* = 1.16), and feedback (*F*(3, 1564) = 46.2, *p* < 0.001, η_p_^2^ = 0.08; see Fig. 3b). The results indicate that mental health practitioners are more hesitant to learn about and use AI-enabled tools that are more patient-centered compared to more therapist-centered tools that have a less direct influence on decisions that affect patients.

### Learning and use intentions across different occupational and demographic groups

Learning and use intentions differed across occupations, with psychiatrists reporting significantly higher intentions to learn (*F*(4, 387) = 4.87, *p* = 0.002, η_p_^2^ = 0.04) and use AI-enabled technologies compared to psychotherapists in training, psychotherapists, and clinical psychologists (*F*(4, 387) = 4.52, *p* = 0.001, η_p_^2^ = 0.04; see Table A1 in the online Supplementary Material 2). All other differences were non-significant (*p* > 0.05). Male practitioners showed higher learning intentions (*t*(153.39) = 2.95, *p* = 0.004, d = 4.17) and use intentions compared to female practitioners (*t*(134.73 = 3.02, *p* = 0.003, d = 3.45; see Table A1 in the online Supplementary Material 2). German practitioners reported significantly lower learning intentions compared to their US counterparts, *t*(363.55) = − 4.03, *p* < 0.001, d = 4.57), however, surprisingly, their use intentions did not differ significantly (*p* > 0.05).

### SEM

For all variables used in the SEM models, means, standard deviations, and correlations can be found in Table A2 in the online Supplementary Material 2. Across all four application areas, the complete models showed better fit indices than the parsimonious models, indicating that the model variables were sufficiently distinct (see Table A3 in the online Supplementary Material 2). In all models, one item from the technology self-efficacy scale had standardized factor loadings below 0.40 and was therefore excluded [75]. The measurement model of the initially proposed model showed only a partially acceptable fit. Therefore, a second version was calculated, which included the correlated error terms for the two reversed-worded items of the ATI scale. Correlating the measurement errors did not significantly alter the parameter estimates of the underlying measurement model. Table 3 shows the fit indices for each of the final models. The model fit indices for RMSEA (≤ 0.056) and SRMR (≤ 0.063) are acceptable to good. The CFI and TFI close to 0.9 are considered marginal levels [76]. As the cutoff-levels for the goodness-of-fit indices depend on model characteristics, such as the sample size and number of variables [77], the complexity of the model and rather small sample size might be the reasons for the CFI and TLI just below the threshold [78].

**Table 3:** Goodness-of-fit indices for each model for each application area

	𝜒^2^ (df)	RMSEA	SRMR	CFI	TLI
Model 1 – Diagnostic	1782.87 (806)	0.056	0.062	0.892	0.879
Model 2 – Treatment	1773.36 (806)	0.056	0.062	0.893	0.880
Model 3 – Feedback	1778.71 (806)	0.056	0.062	0.893	0.880
Model 4 – Management	1767.75 (806)	0.055	0.061	0.894	0.881

RMSEA and SRMR < 0.08; CFI and TLI < 0.9

**Fig. 4:**
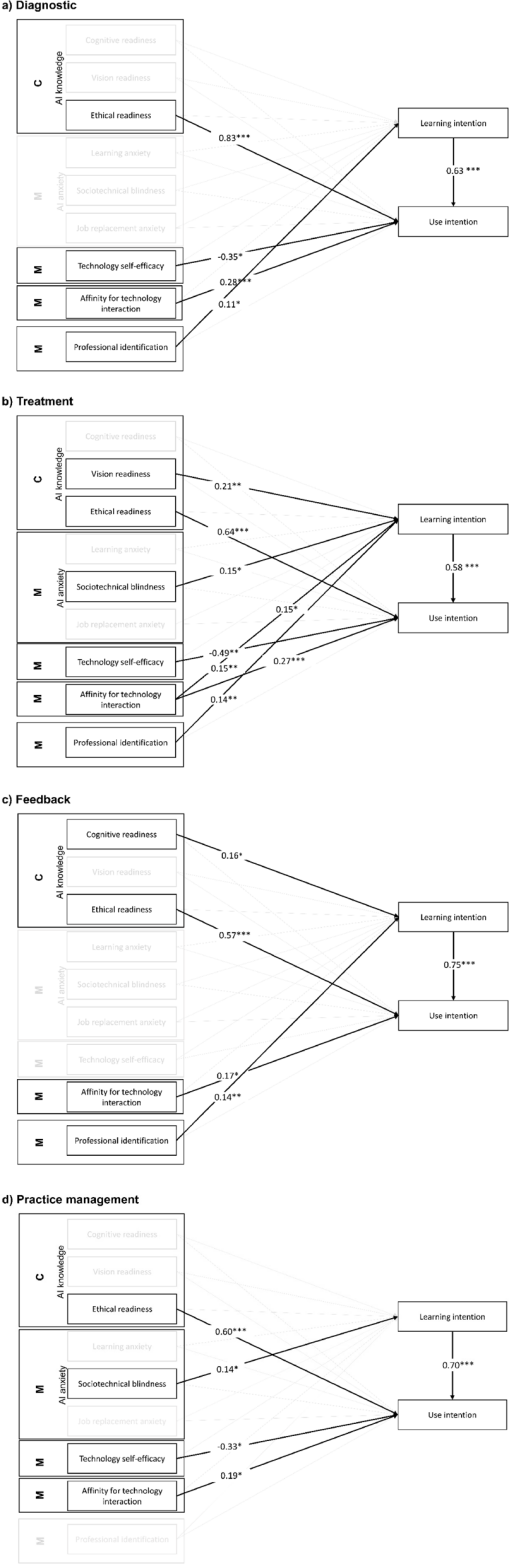
Final structural equation models for **a** diagnostic, **b** treatment, **c** feedback, and **d** practice management tools. Only nonzero paths are displayed. Components of the COM-B model [40] are abbreviated as followed: C = Capability, M = Motivation. * *p* ≤ 0.05; ** *p* ≤ 0.01, *** ≤ 0.001

The results of the final SEM models are presented in Tables A4– A7 in the online Supplementary Material 2. All significant paths are highlighted in Fig. 4. Table 4 shows the SEM results across all four application areas. Across the four models, the control variables alone explained 2.2–4.8% of the variance in learning intentions and 1.3–5.1% in use intentions, and the predictor variables accounted for 46.7–61.0% of the variance in learning intentions and 8.1–17.0% in use intentions. Overall, the relations to use intentions are quite robust while they differ more across the different application areas for learning intentions. Across all application areas, the intention to learn about AI-enabled technologies was positively associated with the intention to use these technologies, supporting H1 for each model. Some paths for the subconstructs of medical artificial intelligence readiness, AI anxiety, beliefs about technological capabilities and professional identity were also relevant across all application areas, however, others differed for each application area (see Table A4– A7 in the online Supplementary Material 2).

**Table 4:** Structural equation modelling results across all four application areas

	Diagnostic				Treatment				Feedback				Management			
	β	[95-CI]	β	95-CI	β	95-CI	β	95-CI	β	95-CI	β	95-CI	β	95-CI	β	95-CI
LI - UI	0.625	[0.54,0.71]			0.577	[0.50, 0.65]			0.749	[0.68, 0.81]			0.698	[0.64, 0.76]		
*Effects*	*LI*		*UI*		LI		UI		LI		UI		LI		UI	
CR	0.050	[− 0.12, 0.22]	0.023	[− 0.19, 0.24]	− 0.074	[− 0.23, 0.09]	0.127	[− 0.10, 0.35]	0.159	[0.01, 0.31]	0.104	[− 0.13, 0.34]	− 0.023	[− 0.16, 0.11]	0.089	[− 0.12, 0.30]
VR	0.125	[− 0.02, 0.27]	− 0.055	[− 0.24, 0.13]	0.205	[0.07, 0.34]	− 0.021	[− 0.21, 0.17]	0.016	[− 0.11, 0.15]	− 0.124	[− 0.33, 0.08]	0.036	[− 0.08, 0.15]	− 0.081	[− 0.26, 0.10]
ER	− 0.039	[− 0.28, 0.20]	0.834	[0.49, 0.17]	− 0.029	[− 0.25, 0.19]	0.635	[0.31, 0.96]	− 0.082	[− 0.29, 0.12]	0.567	[0.23, 0.90]	0.030	[− 0.15, 0.21]	0.599	[0.29, 0.90]
LA	− 0.022	[− 0.11, 0.07]	− 0.039	[− 0.16, 0.08]	− 0.043	[− 0.13, 0.04]	− 0.017	[− 0.14, 0.10]	− 0.040	[− 0.12, 0.04]	− 0.020	[− 0.15, 0.11]	− 0.062	[− 0.13, 0.01]	0.013	[− 0.10, 0.13]
JA	− 0.050	[− 0.21, 0.11]	− 0.009	[− 0.22, 0.20]	− 0.057	[− 0.21, 0.10]	− 0.063	[− 0.28, 0.16]	− 0.060	[− 0.21, 0.09]	0.056	[− 0.18, 0.29]	− 0.116	[− 0.25, 0.18]	0.042	[− 0.17, 0.25]
SB	0.108	[− 0.04, 0.25]	0.010	[− 0.18, 0.20]	0.153	[0.02, 0.29]	0.038	[− 0.15., 0.23]	0.101	[− 0.03, 0.23]	− 0.056	[− 0.16, 0.15]	0.139	[0.02, 0.12]	− 0.050	[− 0.23, 0.13]
TSE	− 0.138	[− 0.39, 0.11]	− 0.350	[− 0.67, − 0.03]	− 0.142	[− 0.38, 0.10]	− 0.489	[− 0.83, − 0.15]	− 0.135	[− 0.36, 0.09]	− 0.205	[− 0.55, 0.14]	− 0.021	[− 0.22, 0.18]	− 0.334	[− 0.65, − 0.02]
ATI	0.089	[− 0.03, 0.21]	0.280	[0.13, 0.46]	0.153	[0.04, 0.27]	0.270	[0.11, 0.43]	0.010	[− 0.10, 0.12]	0.172	[0.00, 0.34]	0.021	[− 0.07, 0.12]	0.186	[0.03, 0.34]
PI	0.113	[0.01, 0.22]	− 0.035	[− 0.17, 0.10]	0.139	[0.04, 0.24]	0.006	[− 0.13, 0.14]	0.142	[0.05, 0.24]	− 0.031	[− 0.18, 0.11]	0.081	[0.00, 0.16]	− 0.038	[− 0.17, 0.09]

β = standardized estimate, 95-CI = 95% confidence interval for the standardized estimate. *LI* Learning intention, *UI* Use intention, *CR* Cognitive readiness, *VR* Vision readiness, *ER* Ethical readiness, *LA* Learning anxiety, *JA* Job replacement anxiety, *SB* Sociotechnical blindness, *TSE* Technology self-efficacy, *ATI* Affinity for technology interaction, *PI* Professional identification

Regarding AI knowledge, cognitive readiness (H2a) was positively associated with the learning intention of the feedback tool, vision readiness (H2b) with the learning intention of the feedback tool, and ethical readiness (H3c) with the use intention across each application area. For the automatic motivational factor AI anxiety, sociotechnical blindness (H2e) demonstrated a positive relationship with the learning intentions of the treatment and practice management tool. For reflective motivational factors, technology self-efficacy (H3g) was negatively related to the use intentions for the diagnostic, treatment, and practice management tool. Further, practitioners’ affinity for technology interaction showed a consistent positive link with the use intentions for all application areas, supporting H3 h for each model. Lastly, professional identification (RQ5) was positively associated with the learning intention for the diagnostic, treatment, and feedback tool.

Controlling for age, gender, and personality did not substantially affect the models for treatment and feedback tools. For the diagnostic tool, the association between professional identification and learning intention, and for practice management tools, the association between cognitive readiness and learning intention were no longer significant (see Table A8– A11 in the online Supplementary Material 2).

## Discussion

Amidst the increasing integration of AI-enabled technologies in healthcare, the present study investigated mental health practitioners’ understanding and familiarity across different application areas for AI-enabled support tools in mental healthcare. Additionally, we examined factors influencing the intention to learn and use AI-enabled technologies across the different areas.

### Current familiarity gaps among mental healthcare professionals

Our study reveals a limited understanding of AI-enabled technologies and significant gap in mental health practitioners’ familiarity with AI-enabled tools for mental health, with nearly half of the surveyed practitioners unaware of these technologies. This low familiarity indicates that many professionals are not informed about the development and potential clinical applications of AI in mental healthcare. Additionally, practitioners primarily gained information through mainstream media such as social media or newspaper articles and less than one-tenth of practitioners who had heard about AI technologies received formal education on the topic, a trend consistent with prior research [79]. Furthermore, the present findings align with an international survey of psychiatrists, which found that less than a quarter had received formal technology training [80]. Adding to the literature, the fact that the majority of our participants were psychotherapists currently enrolled in training suggests that current training programs may not adequately cover AI-related topics, thereby limiting practitioners’ exposure and understanding. As a lack of training and instructions on technology use in healthcare further contributes to an unsafe work environment and medical errors [25], the results underline the need of adjusting the training to emerging technologies.

### Professionals’ varying adoption intentions and application-specific hesitation

The surveyed practitioners were more inclined towards learning rather than actively using AI-enabled technologies in their clinical practice. This supports existing literature indicating that learning and use intentions represent different levels of engagement with technology [27, 28]. For the more practical level of intending to use technologies, practitioners’ main concerns regarding AI technologies, including the lack of transparency of model predictions, data privacy, cyber security, and patient safety [45], might have contributed to their greater use hesitation. Besides, awareness of the need to inform patients about the use of AI technologies in psychotherapeutic decisions and obtain their consent [81, 82], along with understanding how these issues affect their work and patients, might contribute to lower usage intentions.

Moreover, participants demonstrated different levels of willingness to engage with AI-enabled technologies across the application areas. Notably, they were less hesitant towards clinician-centered feedback or practice management tools compared to patient-centered tools, aligning with previous findings [20, 83]. This may be attributed to the higher stakes associated with using technology to inform diagnosis or treatment decisions compared to receiving feedback or administrative support as diagnostic or treatment errors can have severe negative consequences, potentially resulting in wrong or delayed treatment and a worse prognosis [84, 85].

Additionally, our results revealed profession-specific differences, with psychiatrists demonstrating higher learning and use intentions compared to psychotherapists and clinical psychologists. This difference might stem from the specific characteristics of education and work in each occupation. Psychiatrists undergo medical training that already integrates AI-enabled technologies into the curricula, albeit with a focus on other specialties [86]. However, their greater exposure to clinical technologies and closer connection to the broader medical field, where AI use is more prevalent than in psychology, might contribute to their higher adoption intentions. Additionally, since medical prescription are part of psychiatrists’ daily tasks and this area holds widespread potential for AI utilization (for instance see [11]), it might be more natural for them to envision using AI into their practice. The practices of psychotherapists and clinical psychologists in turn are centered more around interpersonal treatment and the patient-therapist relationship [87]. In this context, technology is often perceived not as a substitute for human care [83], hence, it may be challenging for psychotherapists to envision the integration of AI technology into their professional practice, possibly leading to their greater hesitation.

### Individual-level predictors of AI adoption intentions

We found a robust association between the intention to learn and use AI-enabled technologies across all application areas. This aligns with results showing that the willingness to engage in training enhances professionals’ intention to use AI technologies [26]. Consequently, willingness to learn is a first step in engaging with AI technologies and understanding the predictors for both learning and use intention is important. Notably, it is possible, that the difference in explained variance between learning and use intentions may result from the limited familiarity and experience with AI technology. As engagement with AI is a rather gradual process, individuals first need to build familiarity before transitioning to actual use. As a result, learning intention, which is considered a less practical level [27], may be shaped more strongly by motivational factors of less familiar and experienced individuals, with use intention potentially remaining constrained by the lack of prior exposure and the higher stakes of actual implementation.

First, regarding *AI knowledge*, the domain ethical readiness emerged as a significant predictor for use intentions across all application areas, making it a driving force for the intention to use AI-enabled technologies in healthcare. This is in line with research showing that AI ethics awareness was positively correlated with the use intention of AI-based technology in nursing care [51]. The consistent link across all application areas may be explained by the high value of ethics in mental health. Besides general medical ethics, it encompasses elements such as the emotional therapist-patient relationship and handling highly sensitive information, requiring strict adherence to ethical standards [88].

However, learning intentions were influenced differently depending on the application area. On the one hand, the ability to anticipate the technology’s potential impact, involving a deeper understanding of the technologies’ strengths and weaknesses (vision readiness), was positively associated with the intention to learn about treatment support tools. As practitioners were most familiar with treatment tools, it is not surprising that practitioners with a more nuanced understanding are more likely to deepen their knowledge in tools they are already familiar with, likely aiming to refine their knowledge. On the other hand, the basic understanding about AI technologies (cognitive readiness) was positively associated with the intention to learn about feedback tools which practitioners were least familiar with. Practitioners with a basic understanding are therefore eager to explore less familiar tools, potentially driven by curiosity and a desire to broaden their knowledge. Hence, the findings suggest that learning intentions vary based on different facets of practitioners’ AI knowledge, with a basic knowledge leading to a higher intention to learn about new tools and advanced knowledge driving deeper exploration of known tools. These study findings on AI knowledge might help to understand the mixed results found in prior literature which showed a positive association with general AI knowledge in some cases [20, 43], but not in others [45]; while the present study shows that different facets of AI knowledge have varying influences on the adoption intentions for different tools.

Second, none of the subdimensions of *AI anxiety* was associated with use intentions for any application area, contrary to prior findings indicating that AI anxiety impedes AI adoption [20, 50, 51]. However, previous research concentrated on general AI anxiety, without specifically addressing its nuanced facets [20, 50, 51]. For instance, looking at the subdimension of job replacement anxiety, the only moderate levels reported by our participants (see Table A2) might have contributed to this result, indicating that they do not view AI as a threat to their profession. This finding aligns with research indicating that only 4% of psychiatrists believe that future technology will make their jobs obsolete [4, 83]. However, anxiety arising from the belief that AI systems operate without human supervision (sociotechnical blindness) was positively associated with the intention to learn about two AI-enabled application areas: treatment and practice management tools. Contrary to high levels of anxiety, moderate anxiety, as in our study, can have a positive effect on the learning motivation [89] and this might explain the effect in the opposite direction. The effect might have emerged particularly for these two areas, as they are the ones practitioners are most eager to learn about and, in the case of practice management tools, intend to use. Given the pivotal role of human oversight in successfully implementing AI technology, which requires a certain level of tool understanding to monitor its actions and decisions [90–92], practitioners may be more inclined to learn about AI technologies they see themselves engaging with, aiming to equip themselves for ensuring proper oversight if needed.

Third, reflective motivational processes played a pivotal role in both learning and use intentions. Across three application areas (diagnostic, treatment, and practice management), professionals’ *technology self-efficacy* was negatively associated with the intention to use diagnostic, treatment, and practice management tools. However, we found a significant positive correlation between technology self-efficacy and the overall use intention (see Table A2). This discrepancy suggests a suppression effect within the models. This effect occurs when there are multiple predictors in the model, and the overall predictive power of the model is improved by the inclusion of additional predictors that uncover different associations compared to when solely considering technology self-efficacy [93]. Consequently, the association between technology self-efficacy and the use intention is hard to interpret. However, the suppression effect indicates that while technology self-efficacy is negatively associated with the use intention for some application areas, its overall positive correlation with the intention to use suggests that practitioners with higher beliefs in their ability to effectively perform technologically advanced tasks are more inclined to use AI-enabled technologies, which aligns with existing literature [51, 53–56].

Fourth, *affinity for technology interaction*, characterized by the enjoyment and comfort in interacting with technology, showed a positive relationship with the use intention for each tool category. This result was expected based on research from broader hospital settings and other medical domains demonstrating this positive association [59, 60]. From a behavioral perspective, cross-situational consistency may explain this finding as people often maintain behavior across similar contexts [94]. One’s overall positive perception in interacting with technologies might therefore be also transferable to their engagement with technologies at work.

Finally, a strong *professional identity* exhibited a positive association with intentions to learn about three application areas (diagnostic, treatment, and feedback). The non-significant association with the use of learning intention for the practice management tools may relate to the fact that practitioners do not see administrative tasks as closely related to their identity as mental healthcare professionals. The positive association contributes to existing literature by extending prior insights from general healthcare contexts into mental healthcare [63, 65, 95]. Professional identity is a dynamic concept shaped by various factors, including technology implementation [96, 97], and prompting (professionals like) mental healthcare worker to continually assess alignment with evolving work contexts [98, 99]. Despite limited awareness of these technologies, strong identification with their mental health role might motivate them to learn about technologies, facilitating adaption to workplace changes and alignment with their professional identity.

### Limitations and future research

Several limitations should be considered when interpreting the findings of this study. First, the brevity of responses to the open-ended questions may stem from a lack of motivation or time constraints. It is plausible that practitioners possess a more extensive understanding than was conveyed within their response. Future studies could encourage participants to elaborate, for instance by follow-up interviews designed to gather more information on their understanding or by using more objective measures. Second, the inclusion of control variables resulted in the non-significance of vision readiness and professional identification on learning intentions in two models. This, together with the suppression effect on self-efficacy, underscores the complexity of the predictors’ associations and highlights the need for further exploration to understand the nuanced interplay of variables influencing the learning intentions of AI-enabled technologies. Third, no causal relationships could be observed and tested as the present study was cross-sectional. In the future, longitudinal and experimental designs should be employed. Fourth, the data for this study was collected towards the end of 2023, and given the rapid pace of AI development, studies on AI acceptance may not always fully reflect the latest advancements. Future research should continue to account for ongoing technological developments and their evolving impact on AI acceptance. Fifth, the order in which the four AI-enabled application areas were described was not balanced. However, all four application areas were presented to each participant, and the descriptions of the application areas differed substantially, with each description introducing a completely new area. Balancing the order of presentation could be addressed in future research to enhance the robustness and generalizability of the findings. Lastly, participants only got concise descriptions of the different AI application areas without the opportunity for direct practical interaction with the technologies. This might have restricted participants’ depth of understanding and influenced their responses. Future research should explore using detailed, comprehensive, and interactive representations of AI decision-making processes and technologies [100, 101].

### Practical implications

The fact that half of the practitioners have not heard about AI-enabled technology in mental healthcare demonstrates the need for formal education on this topic. The integration of modules on AI-enabled technologies into curricula and professional training programs holds the potential to redirect professional educational frameworks towards future-oriented challenges like technology interaction. Better training regarding the use of technology might prevent medical errors, as research has shown that healthcare practitioners view a lack of technology training as a major cause of errors [25]. Taking it a step further, our study results can also contribute to the development of successful educational frameworks. For instance, ethical knowledge seemed highly relevant for use intentions, hence, education on ethical standards required for technology use is one starting point to ensure their safe and responsible use. As highlighted by Katznelson and Gercke [102], incorporating AI ethics into healthcare training programs is crucial to prepare healthcare professionals for the ethical complexities accompanying AI implementation. Additionally, since affinity for technology interaction was consistently associated with use intentions, the comfort of interacting with technology should also be fostered via practical experiences and on-the-job training. Moreover, addressing hesitations early on or helping users overcome them could involve considering predictors not only in the design of training programs but also the technology itself. One potential solution could involve ensuring more actively that the technology utilizes health data in accordance with legal and ethical norms. Although regulations such as the MDR (Medical Device Regulation) and AIA (Artificial Intelligence Act) are already in place [103], transparently displaying the underlying norms to end users can simultaneously advance their ethical knowledge and ensure adherence to ethical principles. With this, developers can better serve practitioners’ needs and facilitate their adoption of AI technologies in mental healthcare.

## Conclusion

Our study reveals a substantial gap in mental healthcare professionals’ familiarity of AI-enabled technologies in their field. It further underscores the nuanced perception of the different application areas, emphasizing the necessity to consider not only the specific AI application area but also the characteristics of different mental health professionals during the implementation process. Recognizing the pivotal role of learning in initiating engagement, our study suggests that cultivating such engagement via tailored training programs considering robust factors like individuals’ ethical knowledge and affinity for technology interaction could subsequently enhance professionals’ inclination towards utilizing these novel technologies. Moving forward, addressing important factors for each application area will be crucial for the safe integration of AI technologies into mental healthcare practices. Doing so will help bridge the gap between the increasing demand for mental healthcare and limited available therapeutic resources, ultimately improving the accessibility and effectiveness of mental health services.

## Supplementary Information

Supplementary Material 1.

Supplementary Material 2.

## Data Availability

Additional supporting information can be found in the online appendices and on OSF (https://osf.io/9jxwy/).

## Abbreviations

AI: Artificial Intelligence
AIA: Artificial Intelligence Act
ANOVA: Analysis of Variance
CFA: Confirmatory factor analyses
CFI: Comparative Fit Index
H: Hypothesis
MDR: Medical Device Regulation
RMSEA: Root-Mean-Square Error of Approximation
RQ: Research Question
SEM: Structural Equation Modeling
SRMR: Standardized Root-Mean-Square Residual
TLI: Tucker Lewis Index
