# Supplementary material for: Mental health practitioners’ perceptions and adoption intentions of AI-enabled technologies: an international mixed-methods study
Source: BMC Health Serv Res. 2025 Apr 16;25:556. doi: 10.1186/s12913-025-12715-8 (PMC12001504; doi:10.1186/s12913-025-12715-8)
Supplement: Supplementary file 1 — Supplementary Material 1. [file 12913_2025_12715_MOESM1_ESM.pdf]

Intro

Welcome to our study on artificial intelligence (AI) in psychotherapy/ psychiatry!

Aim of the study: The study examines perceptions of AI-enabled technologies in mental health care.

Who may participate: Psychotherapists/Counselors in training, psychotherapists/counselors, psychiatry resident/psychiatrists and clinical

psychologists can participate. Participants must be at least 18 years old.

Study procedure: After you have agreed to participate in the study, you will receive a questionnaire in which your experience and knowledge of AI-enabled technologies in psychotherapy/psychiatry will be assessed. Subsequently, you will be introduced to different AI-enabled tools and we will ask you to answer short questions about these tools. At the end of the study, in addition to demographic data, items measuring technology self-efficacy, affinity for technology, medical artificial intelligence readiness, personality, AI-related anxiety and the intention to learn and use those tools will be collected. Filling in the questionnaire takes approximately **10-15 minutes**.

Your benefits: By participating in the survey, you have a chance to win one of five **$50 Amazon gift cards**.

How we will use the data: The data from this survey will be collected and used for scientific purposes only. Access to the raw data is exclusively by the research team at UKR and LMU Munich. There will be no transfer of data to external parties or to recipients in third countries. The data will be deleted after a retention period of 10 years. Results are published anonymously, i.e. the data cannot be linked to a specific person. However, identification is not

intended even if, in exceptional cases, your identity could be deduced from the data. The fully anonymized data from this study will be made available in a data repository called the Open Science Framework. This study follows the recommendations of the German Research Foundation (DFG) for quality assurance in research.

Data protection: The legal basis for data processing is your consent according to Art. 6 (1) (a) and Art. 9 (2) (a) DSGVO, which you give us together with your answer to the questionnaire. The entity responsible for data processing is: University Hospital Regensburg, Franz-Josef-Strauß-Allee 11, 93053 Regensburg.

Can I revoke my consent? Your participation in this examination is voluntary. You can revoke your consent in writing at any time without giving reasons and without any disadvantage to you. If you revoke your consent, no further data will be collected. However, the data processing that took place until the revocation remains lawful. In the event of revocation, you can also request the deletion of your data. However, the deletion is only possible if the data can still be assigned to your person.

What other rights do I have in relation to data protection? You have the

right to request information from the person responsible for the study (including the provision of a copy of the data free of charge). You can also request the correction of inaccurate data and, if necessary, a transfer of the data you have made available and the restriction of their processing. Please get in touch with: Julia Cecil, LMU Munich (julia.cecil@psy.lmu.de). If you have any questions regarding data protection, you are also welcome to contact the UKR data protection officer: Dr. Wolfgang Börner, Franz Josef-Strauß Allee 11, 93035 Regensburg, dsb@ukr.de, Tel: +49941 944-0 Furthermore, there is a right of appeal to any supervisory authority. Responsible for the UKR is: The Bavarian State Commissioner for Data Protection (poststelle@datenschutz-bayern.de, P.O. Box 22 12 19, 80502 Munich, Tel: +4989 212672-0).

Contact: Julia Cecil, LMU Munich (julia.cecil@psy.lmu.de)

Demographics

Which professional group do you belong to?

Psychotherapist/Counselor in training (e.g. before National Counselor Examination)

Licensed psychotherapist/counselor

Psychiatry resident/Psychiatrist

Clinical psychologist

Other, namely

Which approach to psychotherapy do you practice?

(multiple selection possible)

- (Cognitive) behavioral therapy
- Psychodymic therapy
- Psychoanalytic therapy
- Systemic psychotherapy
- Other, namely

In which institutions are you working?

(multiple selection possible)

Practice

Private practice

General hospital (e.g. psycho-oncology)

Specialized hospital for psychiatry, psychotherapy, psychosomatic medicine or neurology

Rehabilitation clinic

(University) outpatient clinic

Community mental health center/ counseling center

Other, namely

Please indicate the duration of your professional experience

Years

Months

How old are you (in years)?

What gender do you identify with?

- Male
- Female
- Non-binary/ Third gender
- Prefer not to say

The study investigates AI-enabled technologies in psychotherapy/psychiatry among the previously shown professional groups. As you unfortunately do not belong to one of them, we have to terminate your participation in the study early.
If you have mistakenly selected the wrong option, feel free to restart the survey.

Experience

Describe in a few sentences what you understand by **AI**[1]**-enabled**

technologies in the field of psychotherapy/psychiatry and how they could be used in your daily therapeutic work.

---

[1]AI = artificial intelligence

AI refers to computer programs and systems that can perform tasks that normally require human intelligence by analyzing data, recognizing patterns, and learning on their own.

What is your level of experience with AI-enabled technologies in the field of psychotherapy/psychiatry?

- I have never heard of AI-enabled technologies in psychotherapy/psychiatry.
- I have heard of AI-enabled technologies in psychotherapy/psychiatry.
- I have heard of and actively researched AI-enabled technologies in the field of psychotherapy/psychiatry.

In what context have you heard of AI-enabled technologies in psychotherapy/psychiatry?

In what context did you informed yourself about AI-enabled technologies in psychotherapy/psychiatry?

I have informed myself independently (e.g., online, ...).

I attended voluntary information sessions on AI-enabled technologies in psychotherapy/psychiatry.

I have participated in trainings on this topic (e.g., to get training points).

Have you used AI-enabled technologies in psychotherapy/psychiatry in your clinical practice?

Yes

No

Tools

In the following, you will be presented different AI-enabled applications in psychotherapy/psychiatry that are currently under development or already

available on the market. These can be roughly divided into four areas of application.

Please read the information carefully in each case and try to understand the applications. Afterwards, you will be asked questions about the applications.

Application area 1: Screening and diagnostics

Here, AI-based methods are used to screen or diagnose mental disorders. This can be done, for example, by analyzing the patient's speech, voice, facial expressions or other patient data.

Example: Speech software for a more differentiated determination of the severity of the patient's depression.

To what extent do you agree or disagree with the following statements?

	Strongly disagree	Somewhat disagree	Neither agree nor disagree	Somewhat agree	Strongly agree
I intend to learn about AI technologies in the field of screening and diagnostics.	○	○	○	○	○
I intend to use AI technologies in the field of screening and diagnostics in my work.	○	○	○	○	○

Application area 2: Intervention and treatment

Here, AI-enabled methods are used to support interventions and treatments and/or enable (personalized) therapy and/or intervention recommendations.

Example: *Algorithmic analysis of biological markers for the selection of psychotropic drugs individually tailored to the patient.*

To what extent do you agree or disagree with the following statements?

	Strongly disagree	Somewhat disagree	Neither agree nor disagree	Somewhat agree	Strongly agree
I intend to learn about AI technologies in the field of intervention and treatment.	○	○	○	○	○
I intend to use AI technologies in the field of intervention and treatment in my work.	○	○	○	○	○

Application area 3: Feedback for practitioners

Here, AI-enabled methods are used to provide practitioners with feedback on their therapeutic work (especially conversational skills).

Example: *Software that analyzes audio recordings of therapy sessions and produces a report on strengths (e.g. optimal use of reflections) and suggestions for improvement (e.g. more open questions).*

To what extent do you agree or disagree with the following statements?

	Strongly disagree	Somewhat disagree	Neither agree nor disagree	Somewhat agree	Strongly agree
I intend to learn about AI feedback technologies.	○	○	○	○	○
I intend to use AI feedback technologies in my work.	○	○	○	○	○

Application area 4: Practice management/organization

Here, AI-supported methods are used to automate administrative tasks and practice management.

Example: Automated processing of inquiries (e.g. frequently asked questions, appointments) or automated integration of audio recordings of sessions into medical records.

To what extent do you agree or disagree with the following statements?

	Strongly disagree	Somewhat disagree	Neither agree nor disagree	Somewhat agree	Strongly agree
I intend to learn about AI technologies in the field of practice management/ organization.	○	○	○	○	○
I intend to use AI technologies in the field of practice management/ organization in my work.	○	○	○	○	○

Med AI Readiness

To what extent do you agree or disagree with the following statements?

	Strongly disagree	Somewhat disagree	Neither agree nor disagree
I can define the basic concepts of data science	○	○	○
I can define the basic concepts of statistics	○	○	○
I can explain how AI systems are trained	○	○	○
I can define the basic concepts and terminology of AI	○	○	○

To what extent do you agree or disagree with the following statements?

	Strongly disagree	Somewhat disagree	Neither agree nor disagree
I can explain the limitations of AI technology	○	○	○
I can explain the strengths and weaknesses of AI technology	○	○	○
I can foresee the opportunities and threats that AI technology can create in healthcare	○	○	○

To what extent do you agree or disagree with the following statements?

	Strongly disagree	Somewhat disagree	Neither agree nor disagree
I will be able to use health data in accordance with legal and ethical norms	○	○	○
I will be able to conduct under ethical principles while using AI technologies	○	○	○
I will be able to follow legal regulations regarding the use of AI technologies in healthcare	○	○	○

AI Anxiety

To what extent do you agree or disagree with the following statements?

	Strongly disagree	Disagree	Somewhat disagree	Ne ag no dis
Taking a class about the development of AI technologies makes me anxious	○	○	○	
Learning to use AI technologies makes me anxious	○	○	○	
Please click "Agree"	○	○	○	
Learning how an AI technology works makes me anxious	○	○	○	
Learning to use specific functions of an AI technology makes me anxious	○	○	○	
Learning to interact with an AI technology makes me anxious	○	○	○	
Being unable to keep up with the advances associated with AI technologies makes me anxious	○	○	○	

Reading an AI technology manual makes me anxious	○	○	○	
Learning to understand all of the special functions associated with an AI technology makes me anxious	○	○	○	

To what extent do you agree or disagree with the following statements?

	Strongly disagree	Disagree	Somewhat disagree	Ne ag no dis
I am afraid that AI technologies will replace someone's job	○	○	○	
I am afraid that if I begin to use AI technologies I will become dependent upon them and lose some of my reasoning skills	○	○	○	
I am afraid that widespread use of humanoid robots will take jobs away from people	○	○	○	

To what extent do you agree or disagree with the following statements?

	Strongly disagree	Disagree	Somewhat disagree	Ne ag no dis
I am afraid that an AI technology may make us dependent	○	○	○	
I am afraid that an AI technology may make us even lazier	○	○	○	
I am afraid that an AI technology may replace humans	○	○	○	
I am afraid that an AI technology may get out of control and malfunction	○	○	○	
I am afraid that an AI technology may be misused	○	○	○	
I am afraid of various problems potentially associated with an AI technology	○	○	○	
I am afraid that an AI product may lead to robot autonomy	○	○	○	

Self-Efficacy/ Attitude

To what extent do you agree or disagree with the following statements?

	Strongly disagree	Disagree	Neither agree nor disagree	Agree	Stronlgy agree
When I have to learn a new task that is high-tech, my first reaction is that I'm sure I can do it.	○	○	○	○	○
In terms of my ability to learn new tasks that are high-tech, I would describe myself as one of the best in my workgroup.	○	○	○	○	○
In the past, I have had a great amount of experience (either on or off the job) working on high-tech tasks.	○	○	○	○	○
I am extremely confident that I can					

learn to use AI-enabled technologies on my job.	◯	◯	◯	◯	◯
AI-enabled technologies will allow me to perform my job better and more efficiently.	◯	◯	◯	◯	◯

To what extent do you agree or disagree with the following statements?

	Completely disagree	Largely disagree	slightly disagree	slightly agree	largely agree	Completely agree
I like to occupy myself in greater detail with technical systems.	◯	◯	◯	◯	◯	◯
I like testing the functions of new technical systems.	◯	◯	◯	◯	◯	◯

It is enough for me that a technical system works, I don’t care how or why.						
It is enough for me to know the basic functions of a technical system						

To what extent do you agree or disagree with the following statements?

	Strongly disagree	Disagree	Somewhat disagree	Neither agree nor disagree	Somewhat agree	Agree	Str agr
In general, when someone praises psychotherapists/ counselors in training, it feels like a personal compliment.							
In general, when someone criticizes psychotherapists/ counselors in training, it feels like a personal insult.							
When I talk about psychotherapists/							

counselors in training, I usually say “we” rather than “they.”	○	○	○	○	○	○	(
Psychotherapy’s successes are my successes.	○	○	○	○	○	○	(
If a story in the media criticized psychotherapists/ counselors in training, I would feel embarrassed.	○	○	○	○	○	○	(

To what extent do you agree or disagree with the following statements?

	Strongly disagree	Disagree	Somewhat disagree	Neither agree nor disagree	Somewhat agree	Agree	Str agı
In general, when someone praises							

licensed psychotherapists/ counselors, it feels like a personal compliment.							
In general, when someone criticizes licensed psychotherapists/ counselors, it feels like a personal insult.							
When I talk about licensed psychotherapists/ counselors, I usually say "we" rather than "they."							
Psychotherapy's successes are my successes.							

To what extent do you agree or disagree with the following statements?

	Strongly disagree	Disagree	Somewhat disagree	Neither agree nor disagree	Somewhat agree	Agree	Stro[illegible] agre[illegible]
If a story in the media criticized [illegible]							
In general,							

licensed psychotherapists/ counselors, I would feel embarrassed.	○	○	○	○	○	○	○
when someone praises psychiatrists, it feels like a personal compliment.	○	○	○	○	○	○	○
In general, when someone criticizes psychiatrists, it feels like a personal insult.	○	○	○	○	○	○	○
When I talk about psychiatrists, I usually say "we" rather than "they."	○	○	○	○	○	○	○

To what extent do you agree or disagree with the following statements?

	Strongly disagree	Disagree	Somewhat disagree	Neither agree nor disagree	Somewhat agree	Agree	Stro[illegible] agre[illegible]
Psychotherapy's successes are my successes.	○	○	○	○	○	○	○
In general, when someone praises clinical psychologists, it feels like a personal [illegible]	○	○	○	○	○	○	○
If a story in the media criticized psychiatrists, I would feel [illegible]	○	○	○	○	○	○	○

embarrassed. compliment.

In general, when someone criticizes clinical psychologists, it feels like a personal insult.	○	○	○	○	○	○	○
When I talk about clinical psychologists, I usually say “we” rather than “they.”	○	○	○	○	○	○	○

Personality

Psychotherapy’s successes are my successes.	○	○	○	○	○	○	○
If a story in the media criticized clinical psychologists, I would feel embarrassed.	○	○	○	○	○	○	○

I see myself as someone who ...

	Strongly disagree	Disagree a little	Neither agree nor disagree	Agree a little
... is reservered	○	○	○	○
... is generally trusting	○	○	○	○
... tends to be lazy	○	○	○	○
... is relaxed, handles stress well	○	○	○	○
Please select "Disagree a little"	○	○	○	○
... has few artistic interests	○	○	○	○
... is outgoing, sociable	○	○	○	○
... tends to find fault with others	○	○	○	○

... does a thorough job	○	○	○	○
... gets nervous easily	○	○	○	○
... has an active imagination	○	○	○	○

End

To what extent do you agree or disagree with the following statement?

	Strongly disagree	Disagree	Neither agree nor disagree	Agree	Strongly agree
In order for AI-enabled technologies to simplify everyday clinical work, something would have to change fundamentally in the psychotherapeutic care landscape.	○	○	○	○	○

What would have to change in your opinion?

Is there anything else you would like to add about this study or to better understand your answers?

Powered by Qualtrics
