# Supplementary material for: Mental health practitioners’ perceptions and adoption intentions of AI-enabled technologies: an international mixed-methods study
Source: BMC Health Serv Res. 2025 Apr 16;25:556. doi: 10.1186/s12913-025-12715-8 (PMC12001504; doi:10.1186/s12913-025-12715-8)
Supplement: Supplementary file 2 — Supplementary Material 2. [file 12913_2025_12715_MOESM2_ESM.docx]

**Online Appendix for:**

**Mental health practitioners’ perceptions and adoption intentions of AI-enabled technologies: an international mixed-methods study**

**Background to the AI Application Areas**

1. **Diagnostics**: AI-enabled methods are used to screen or diagnose mental disorders by analyzing patient data such as speech, voice, or facial expressions.

- Example: Speech software for a more differentiated determination of the severity of the patient's depression.
- Reference: Menne, F., Dörr, F., Schräder, J., Tröger, J., Habel, U., König, A., & Wagels, L. (2024). The voice of depression: speech features as biomarkers for major depressive disorder. BMC Psychiatry 24, 794 (2024). <https://doi.org/10.1186/s12888-024-06253-6>

1. **Intervention and Treatment**: AI-enabled methods support interventions and treatments by enabling (personalized) therapy and intervention recommendations.

- Example: Algorithmic analysis of biological markers for the selection of psychotropic drugs individually tailored to the patient.
- Reference: Lin, E., Lin, C. H., & Lane, H. Y. (2020). Precision psychiatry applications with pharmacogenomics: artificial intelligence and machine learning approaches. International journal of molecular sciences, 21(3), 969. <https://doi.org/10.3390/ijms21030969>

1. **Feedback for Practitioners**: AI-enabled methods provide practitioners with feedback on their therapeutic work, particularly conversational skills.

- Example: Software that analyzes audio recordings of therapy sessions and produces a report on strengths (e.g., optimal use of reflections) and suggestions for improvement (e.g., more open questions).
- Reference: Cummins, R., Ewbank, M. P., Martin, A., Tablan, V., Catarino, A., & Blackwell, A. D. (2019, May). TIM: a tool for gaining insights into psychotherapy. In The World Wide Web Conference (pp. 3503-3506). <https://doi.org/10.1145/3308558.3314128>

1. **Practice Management/Organization**: AI-enabled methods automate administrative tasks and practice management.

- Example: Automated processing of inquiries (e.g., frequently asked questions, appointments) or automated integration of audio recordings of sessions into medical records.
- Reference: Sadeh-Sharvit, S., Camp, T. D., Horton, S. E., Hefner, J. D., Berry, J. M., Grossman, E., & Hollon, S. D. (2023). Effects of an Artificial Intelligence Platform for Behavioral Interventions on Depression and Anxiety Symptoms: Randomized Clinical Trial. Journal of medical Internet research, 25, e46781. <https://doi.org/10.2196/46781>

**Adoption intentions for different occupational and demographic variables**

**Table A1**

*Mean and standard deviations for AI anxiety, learning and use intentions by occupational and demographic variables*

|  | Profession | | | |  | Gender | |  | Country | |
| --- | --- | --- | --- | --- | --- | --- | --- | --- | --- | --- |
|  | Psy | Pth | Pth-T | ClinP |  | *male* | *female* |  | *GER* | *US* |
| LI | 4.11 (0.78) | 3.41 (0.85) | 3.67 (0.85) | 3.55 (1) |  | 3.89 (0.82) | 3.58 (0.88) |  | 3.51 (0.91) | 3.86 (0.79) |
| UI | 3.66 (1.01) | 2.95 (0.76) | 3.12 (0.84) | 3.08 (0.98) |  | 3.40 (0.98) | 3.06 (0.83) |  | 3.17 (0.87) | 3.1 (0.88) |
| *Note*. LI = Learning intention, UI = Use intention, Psy = Psychiatrist, Pth = Psychotherapist, Pth-T = Psychotherapist in training, ClinP = Clinical psychologist, GER = Germany | | | | | | | | | | |

| **Correlations of SEM variables**  **Table A2** | | | | | | | | | | | | | | | | | | |
| --- | --- | --- | --- | --- | --- | --- | --- | --- | --- | --- | --- | --- | --- | --- | --- | --- | --- | --- |
| *Means, standard deviations, and correlations between the variables used in the SEM model.* | | | | | | | | | | | | | | | | | | |
|  | *M (SD)* | 1 | 2 | 3 | 4 | 5 | 6 | 7 | 8 | 9 | 10 | 11 | 12 | 13 | 14 | 15 | 16 | 17 |
| 1. CR | 3.02  (0.93) |  |  |  |  |  |  |  |  |  |  |  |  |  |  |  |  |  |
| 2. VR | 3.14  (0.89) | .50*** |  |  |  |  |  |  |  |  |  |  |  |  |  |  |  |  |
| 3. ER | 3.51  (0.78) | .21*** | .25*** |  |  |  |  |  |  |  |  |  |  |  |  |  |  |  |
| 4. LA | 2.82  (1.38) | -.26*** | -.12* | -.14** |  |  |  |  |  |  |  |  |  |  |  |  |  |  |
| 5. JA | 3.79  (1.37) | -.13* | .01 | -.08 | .47*** |  |  |  |  |  |  |  |  |  |  |  |  |  |
| 6. SB | 4.50  (1.21) | -.13** | .04 | -.13** | .50*** | .65*** |  |  |  |  |  |  |  |  |  |  |  |  |
| 7. TSE | 3.05  (0.75) | .45*** | .33*** | .29*** | -.31*** | -.10 | -.20*** |  |  |  |  |  |  |  |  |  |  |  |
| 8. ATI | 3.17  (1.03) | .47*** | .27*** | .19*** | -.37*** | -.13* | -.20*** | .56*** |  |  |  |  |  |  |  |  |  |  |
| 9. PI | 4.04  (1.12) | .10 | .17*** | .10* | .25*** | .25*** | .15** | .20** | .02 |  |  |  |  |  |  |  |  |  |
| 10. LI | 3.65  (0.88) | .19*** | .17*** | .20*** | -.06 | .01 | .01 | .24*** | .25*** | .10 |  |  |  |  |  |  |  |  |
| Table A2 (continued) | | | | | | | | | | | | | | | | |  |  |
|  | *M (SD)* | 1 | 2 | 3 | 4 | 5 | 6 | 7 | 8 | 9 | 10 | 11 | 12 | 13 | 14 | 15 | 16 | 17 |
| 11. UI | 3.14  (0.88) | .14** | .03 | .27*** | -.12* | -.09 | -.13*** | .23*** | .23*** | -.04 | .73*** |  |  |  |  |  |  |  |
| 12. AGE | 34.34  (10.46) | .03 | .01 | .05 | -.06 | .02 | .09 | .02 | .15** | -.09 | -.05 | .01 |  |  |  |  |  |  |
| 13. GEN | 1.79  (0.48) | -.16** | -.07 | -.13* | .12* | -.04 | .05 | -.25*** | -.27*** | .05 | -.14** | -.15** | -.17*** |  |  |  |  |  |
| 14. OP | 3.81  (0.90) | .13** | .06 | .04 | -.05 | -.04 | -.04 | .02 | .05 | .04 | .06 | .02 | .03 | .01 |  |  |  |  |
| 15. CO | 3.89  (0.76) | .11* | .13** | .09 | -.04 | -.08 | -.04 | .06 | .04 | .05 | -.01 | -.08 | .00 | .16** | .02 |  |  |  |
| 16. EX | 3.40  (0.95) | .06 | -.08 | .02 | -.08 | -.04 | -.03 | .06 | -.00 | -.06 | -.02 | .05 | .02 | -.00 | .17*** | .14** |  |  |
| 17. AG | 3.51  (0.75) | -.04 | -.02 | -.01 | -.11* | -.09 | -.11* | .03 | .01 | -.03 | -.00 | .04 | -.03 | -.00 | .02 | .12* | .10 |  |
| 18. NE | 3.00  (0.97) | -.14** | -.05 | -.09 | .29*** | .18*** | .17*** | -.21*** | -.23*** | .18*** | .04 | -.02 | -.22*** | .17*** | -.03 | -.19*** | -.20*** | -.25*** |

*Note*. *M* = mean. *SD* = standard deviation.
* *p* < .05, ** *p* < .01, *** *p* < .001

CR = Cognitive readiness, VR = Vision readiness, ER = Ethical readiness, LA = Learning anxiety, JA = Job replacement anxiety, SB = Sociotechnical blindness, TSE = Technology self-efficacy, ATI = Affinity for technology interaction, PI = Professional identification, LI = Learning intention, UI = Use intention, AGE = Age, GEN = Gender, OP = Openness, CO = Conscientiousness, EX = Extraversion, AG = Agreeableness, NE = Neuroticis

| **Model fit of the parsimonious models**  **Table A3** | | | | | |
| --- | --- | --- | --- | --- | --- |
| *Goodness-of-fit indices for the three parsimonious models for all application areas* | | | | | |
|  | 𝜒^2^ (df) | RMSEA | SRMR | CFI | TLI |
| Model 1 | 2339.80 (758) | .073 | .074 | .818 | .803 |
| Model 2 | 9514.74 (820) | .086 | .091 | .751 | .731 |
| Model 3 | 2339.80 (758) | .073 | .074 | .816 | .803 |
| *Note.* RMSEA and SRMR < .08; CFI and TLI < .9  Model 1 = combined subscales of readiness for medical AI; Model 2 = combined subscales of AI anxiety; Model 3 = combined affinity for technology interaction and technology self-efficacy. | | | | | |

| **Structural equation model across all four application areas**  **Table A4**  *Structural equation modeling results for the diagnostic tool* | | | | | | | | | | | |  |
| --- | --- | --- | --- | --- | --- | --- | --- | --- | --- | --- | --- | --- |
|  | β | SE | 95-CI | *p* | H/RQ |  | β | SE | 95-CI | *p* | H/RQ |  |
| LI → UI | 0.625 | 0.041 | [.54,.71] | 0.000 | H1 | ✓ |  |  |  |  |  |  |
| *Effects on LI* | |  |  |  |  |  | *Effects on UI* | |  |  |  |  |
| CR | 0.050 | 0.084 | [-.12, .22] | 0.551 | H2a | — | 0.023 | 0.109 | [-.19, .24] | 0.829 | H3a | — |
| VR | 0.125 | 0.072 | [-.02, .27] | 0.082 | H2b | — | -0.055 | 0.093 | [-.24, .13] | .554 | H3b | — |
| ER | -0.039 | 0.122 | [-.28, .20] | 0.747 | H2c | — | 0.834 | 0.173 | [.49, .17] | 0.000 | H3c | ✓ |
| LA | -0.022 | 0.046 | [-.11, .07] | 0.633 | H2d | — | -0.039 | 0.060 | [-.16, .08] | 0.514 | H3d | — |
| JA | -0.050 | 0.083 | [-.21, .11] | 0.547 | H2e | — | -0.009 | 0.108 | [-.22, .20] | 0.937 | H3e | — |
| SB | 0.108 | 0.074 | [-.04, .25] | 0.141 | H2f | — | 0.010 | 0.095 | [-.18, .20] | 0.919 | H3f | — |
| TSE | -0.138 | 0.126 | [-.39, .11] | 0.274 | H2g | — | -0.350 | 0.163 | [-.67, -.03] | 0.032 | H3g | — |
| ATI | 0.089 | 0.062 | [-.03, .21] | 0.156 | H2h | — | 0.280 | 0.079 | [.13, .46] | 0.000 | H3h | ✓ |
| PI | 0.113 | 0.052 | [.01, .22] | 0.031 | RQ5 | ✓ | -0.035 | 0.067 | [-.17, .10] | 0.605 | RQ6 | — |
| *Note*s. LI = Learning intention, UI = Use intention CR = Cognitive readiness, VR = Vision readiness, ER = Ethical readiness, LA = Learning anxiety, JA = Job replacement anxiety, SB = Sociotechnical blindness, TSE = Technology self-efficacy, ATI = Affinity for technology interaction, PI = Professional identification,  ✓ = hypothesis/ research question confirmed; — = hypothesis/ research question not confirmed. | | | | | | | | | | | |  |

| **Table A5**  *Structural equation modeling results for the treatment tool* | | | | | | | | | | | |  |
| --- | --- | --- | --- | --- | --- | --- | --- | --- | --- | --- | --- | --- |
|  | β | SE | 95-CI | *p* | H/RQ |  | β | SE | 95-CI | *p* | H/RQ |  |
| LI → UI | 0.577 | 0.038 | [.50, .65] | 0.000 | H1 | ✓ |  |  |  |  |  |  |
| *Effects on LI* | |  |  |  |  |  | *Effects on UI* | |  |  |  |  |
| CR | -0.074 | 0.081 | [-.23, .09] | 0.364 | H2a | — | 0.127 | 0.113 | [-.10, .35] | 0.262 | H3a | — |
| VR | 0.205 | 0.070 | [.07, .34] | 0.003 | H2b | ✓ | -0.021 | 0.097 | [-.21, .17] | 0.831 | H3b | — |
| ER | -0.029 | 0.112 | [-.25, .19] | 0.792 | H2c | — | 0.635 | 0.164 | [.31, .96] | 0.000 | H3c | ✓ |
| LA | -0.043 | 0.044 | [-.13, .04] | 0.326 | H2d | — | -0.017 | 0.062 | [-.14, .10] | 0.784 | H3d | — |
| JA | -0.057 | 0.080 | [-.21, .10] | 0.476 | H2e | — | -0.063 | 0.112 | [-.28, .16] | 0.572 | H3e | — |
| SB | 0.153 | 0.071 | [.02, .29] | 0.030 | H2f | — | 0.038 | 0.098 | [-.15., .23] | 0.700 | H3f | — |
| TSE | -0.142 | 0.124 | [-.38, .10] | 0.252 | H2g | — | -0.489 | 0.173 | [-.83, -.15] | 0.005 | H3g | — |
| ATI | 0.153 | 0.060 | [.04, .27] | 0.010 | H2h | ✓ | 0.270 | 0.081 | [.11, .43] | 0.001 | H3h | ✓ |
| PI | 0.139 | 0.050 | [.04, .24] | 0.006 | RQ5 | ✓ | 0.006 | 0.070 | [-.13, .14] | 0.930 | RQ6 | — |
| *Notes*. LI = Learning intention, UI = Use intention CR = Cognitive readiness, VR = Vision readiness, ER = Ethical readiness, LA = Learning anxiety, JA = Job replacement anxiety, SB = Sociotechnical blindness, TSE = Technology self-efficacy, ATI = Affinity for technology interaction, PI = Professional identification,  ✓ = hypothesis/ research question confirmed; — = hypothesis/ research question not confirmed. | | | | | | | | | | | |  |

| **Table A6**  *Structural equation modeling results for the feedback tool* | | | | | | | | | | | |  |
| --- | --- | --- | --- | --- | --- | --- | --- | --- | --- | --- | --- | --- |
|  | β | SE | 95-CI | *p* | H/RQ |  | β | SE | 95-CI | *p* | H/RQ |  |
| LI → UI | 0.749 | 0.033 | [.68, .81] | 0.000 | H1 | ✓ |  |  |  |  |  |  |
| *Effects on LI* | |  |  |  |  |  | *Effects on UI* | |  |  |  |  |
| CR | 0.159 | 0.077 | [.01, .31] | 0.039 | H2a | ✓ | 0.104 | 0.120 | [-.13, .34] | 0.383 | H3a | — |
| VR | 0.016 | 0.066 | [-.11, .15] | 0.810 | H2b | — | -0.124 | 0.103 | [-.33, .08] | 0.229 | H3b | — |
| ER | -0.082 | 0.105 | [-.29, .12] | 0.433 | H2c | — | 0.567 | 0.170 | [.23, .90] | 0.001 | H3c | ✓ |
| LA | -0.040 | 0.042 | [-.12, .04] | 0.338 | H2d | — | -0.020 | 0.066 | [-.15, .11] | 0.765 | H3d | — |
| JA | -0.060 | 0.076 | [-.21, .09] | 0.426 | H2e | — | 0.056 | 0.118 | [-.18, .29] | 0.639 | H3e | — |
| SB | 0.101 | 0.067 | [-.03, .23] | 0.130 | H2f | — | -0.056 | 0.104 | [-.16, .15] | 0.586 | H3f | — |
| TSE | -0.135 | 0.144 | [-.36, .09] | 0.237 | H2g | — | -0.205 | 0.178 | [-.55, .14] | 0.248 | H3g | — |
| ATI | 0.010 | 0.055 | [-.10, .12] | 0.853 | H2h | — | 0.172 | 0.086 | [.00, .34] | 0.045 | H3h | ✓ |
| PI | 0.142 | 0.048 | [.05, .24] | 0.003 | RQ5 | ✓ | -0.031 | 0.073 | [-.18, .11] | 0.637 | RQ6 | — |
| *Notes*. LI = Learning intention, UI = Use intention CR = Cognitive readiness, VR = Vision readiness, ER = Ethical readiness, LA = Learning anxiety, JA = Job replacement anxiety, SB = Sociotechnical blindness, TSE = Technology self-efficacy, ATI = Affinity for technology interaction, PI = Professional identification,  ✓ = hypothesis/ research question confirmed; — = hypothesis/ research question not confirmed. | | | | | | | | | | | |  |

| **Table A7**  *Structural equation modeling results for the practice management tool* | | | | | | | | | | | |  |
| --- | --- | --- | --- | --- | --- | --- | --- | --- | --- | --- | --- | --- |
|  | β | SE | 95-CI | *p* | H/RQ |  | β | SE | 95-CI | *p* | H/RQ |  |
| LI → UI | 0.698 | 0.032 | [.64, .76] | 0.000 | H1 | ✓ |  |  |  |  |  |  |
| *Effects on LI* | |  |  |  |  |  | *Effects on UI* | |  |  |  |  |
| CR | -0.023 | 0.067 | [-.16, .11] | 0.731 | H2a | — | 0.089 | 0.109 | [-.12, .30] | 0.414 | H3a | — |
| VR | 0.036 | 0.057 | [-.08, .15] | 0.528 | H2b | — | -0.081 | 0.092 | [-.26, .10] | 0.382 | H3b | — |
| ER | 0.030 | 0.092 | [-.15, .21] | 0.748 | H2c | — | 0.599 | 0.156 | [.29, .90] | 0.000 | H3c | ✓ |
| LA | -0.062 | 0.037 | [-.13, .01] | 0.094 | H2d | — | 0.013 | 0.059 | [-.10, .13] | 0.821 | H3d | — |
| JA | -0.116 | 0.067 | [-.25, .18] | 0.084 | H2e | — | 0.042 | 0.107 | [-.17, .25] | 0.692 | H3e | — |
| SB | 0.139 | 0.059 | [.02, .12] | 0.019 | H2f | — | -0.050 | 0.094 | [-.23, .13] | 0.593 | H3f | — |
| TSE | -0.021 | 0.100 | [-.22, .18] | 0.831 | H2g | — | -0.334 | 0.163 | [-.65, -.02] | 0.040 | H3g | — |
| ATI | 0.021 | 0.049 | [-.07, .12] | 0.669 | H2h | — | 0.186 | 0.078 | [.03, .34] | 0.017 | H3h | ✓ |
| PI | 0.081 | 0.042 | [.00, .16] | 0.051 | RQ5 | — | -0.038 | 0.067 | [-.17, .09] | 0.566 | RQ6 | — |
| *Notes*. LI = Learning intention, UI = Use intention CR = Cognitive readiness, VR = Vision readiness, ER = Ethical readiness, LA = Learning anxiety, JA = Job replacement anxiety, SB = Sociotechnical blindness, TSE = Technology self-efficacy, ATI = Affinity for technology interaction, PI = Professional identification,  ✓ = hypothesis/ research question confirmed; — = hypothesis/ research question not confirmed. | | | | | | | | | | | |  |

| **Structural equation models with control variables**  **Table A8**  *Structural equation modeling results with control variables for the diagnostic tool* | | | | | | | | | | |
| --- | --- | --- | --- | --- | --- | --- | --- | --- | --- | --- |
|  | β | SE | 95-CI | *p* | H | β | SE | 95-CI | *p* | H |
| LI → UI | .635 | 0.042 | [.55,.72] | .000 | H1 |  |  |  |  |  |
| *Effects on LI* | |  |  |  |  | *Effects on UI* | |  |  |  |
| CR | .033 | 0.083 | [-.13, .20] | .691 | H2a | -.022 | 0.108 | [-.21, .21] | .988 | H3a |
| VR | .118 | 0.072 | [-.03, .26] | .103 | H2b | -.010 | 0.094 | [-.20, .18] | .914 | H3b |
| ER | -.087 | 0.126 | [-.33, .16] | .492 | H2c | **.814** | 0.179 | [.46, .17] | **.000** | H3c |
| LA | -.027 | 0.046 | [-.12, .07] | .562 | H2d | -.024 | 0.060 | [-.14, .09] | .695 | H3d |
| JA | -.049 | 0.084 | [-.21, .12] | .558 | H2e | -.009 | 0.109 | [-.22, .20] | .937 | H3e |
| SB | .112 | 0.073 | [-.03, .25] | .125 | H2f | -.013 | 0.094 | [-.20, .17] | .887 | H3f |
| TSE | -.113 | 0.132 | [-.37, .15] | .390 | H2g | **-.445** | 0.172 | [-.78, -.11] | **.010** | H3g |
| ATI | .086 | 0.063 | [-.04, .21] | .168 | H2h | **.284** | 0.080 | [.13, .44] | **.000** | H3h |
| **PI** | .096 | 0.053 | [.01, .20] | **.069** | RQ5 | .016 | 0.069 | [-.12, .15] | .819 | RQ6 |
| AGE | -.004 | 0.004 | [-.01, .00] | .293 |  | -.001 | 0.005 | [-.01, .01] | .841 |  |
| GEN | -.089 | 0.102 | [-.29, .11] | .382 |  | -.266 | 0.131 | [-.52, -.01] | **.043** |  |
| OP | .079 | 0.047 | [-.01, .17] | .091 |  | .002 | 0.061 | [-.12, .12] | .972 |  |
| CO | .090 | 0.058 | [-.02, .20] | .120 |  | -.135 | 0.075 | [-.28, .01] | .072 |  |
| EX | -.055 | 0.046 | [-.15, .04] | .236 |  | .056 | 0.060 | [-.06, .17] | .347 |  |
| AG | .002 | 0.057 | [-.11, .11] | .973 |  | -.007 | 0.074 | [-.15, .14] | .920 |  |
| NE | .076 | 0.047 | [-.02, .17] | .109 |  | -.004 | 0.061 | [-.12, .12] | .951 |  |
| *Note*s. LI = Learning intention, UI = Use intention CR = Cognitive readiness, VR = Vision readiness, ER = Ethical readiness, LA = Learning anxiety, JA = Job replacement anxiety, SB = Sociotechnical blindness, TSE = Technology self-efficacy, ATI = Affinity for technology interaction, PI = Professional identification, AGE = Age, GEN = Gender, OP = Openness, CO = Conscientiousness, EX = Extraversion, AG = Agreeableness, NE = Neuroticism | | | | | | | | | | |

| **Table A9**  *Structural equation modeling results with control variables for the treatment tool* | | | | | | | | | | |
| --- | --- | --- | --- | --- | --- | --- | --- | --- | --- | --- |
|  | β | SE | 95-CI | *p* | H | β | SE | 95-CI | *p* | H |
| LI → UI | **.582** | 0.038 | [.51, .66] | **.000** | H1 |  |  |  |  |  |
| *Effects on LI* | |  |  |  |  | *Effects on UI* | |  |  |  |
| CR | -.079 | 0.082 | [-.24, .08] | .330 | H2a | .117 | 0.114 | [-.11, .34] | .303 | H3a |
| VR | **.187** | 0.071 | [.05, .33] | **.009** | H2b | .011 | 0.099 | [-.18, .21] | .908 | H3b |
| ER | -.033 | 0.117 | [-.26, .20] | .776 | H2c | **.612** | 0.171 | [.28, .95] | **.000** | H3c |
| LA | -.061 | 0.045 | [-.15, .03] | .173 | H2d | -.025 | 0.063 | [-.15, .10] | .688 | H3d |
| JA | -.064 | 0.082 | [-.22, .10] | .434 | H2e | -.074 | 0.114 | [-.30, .15] | .517 | H3e |
| SB | **.168** | 0.071 | [.03, .31] | **.018** | H2f | .019 | 0.098 | [-.18., .21] | .848 | H3f |
| TSE | -.109 | 0.130 | [-.36, .15] | .400 | H2g | **-.507** | 0.183 | [-.87, -.15] | **.006** | H3g |
| ATI | **.146** | 0.060 | [.03, .27] | **.016** | H2h | **.277** | 0.083 | [.12 .44] | **.001** | H3h |
| **PI** | **.120** | 0.052 | [.02, .22] | **.020** | RQ5 | .029 | 0.072 | [-.11, .17] | .689 | RQ6 |
| AGE | -.004 | 0.004 | [-.01, .00] | .288 |  | .004 | 0.006 | [-.01, .02] | .519 |  |
| GEN | -.128 | 0.098 | [-.33, .05] | .159 |  | -.025 | 0.138 | [-.33, .25] | .856 |  |
| OP | .021 | 0.046 | [-.07, .11] | .644 |  | -.048 | 0.064 | [-.17, .08] | .455 |  |
| CO | .091 | 0.056 | [-.02, .20] | .105 |  | -.110 | 0.079 | [-.26, .05] | .161 |  |
| EX | -.030 | 0.045 | [-.12, .06] | .499 |  | .005 | 0.063 | [-.12, .13] | .939 |  |
| AG | -.035 | 0.056 | [-.14, .07] | .530 |  | .007 | 0.078 | [-.15, .16] | .933 |  |
| NE | .061 | 0.046 | [-.03, .15] | .186 |  | .087 | 0.064 | [-.04, .21] | .179 |  |
| *Notes*. LI = Learning intention, UI = Use intention CR = Cognitive readiness, VR = Vision readiness, ER = Ethical readiness, LA = Learning anxiety, JA = Job replacement anxiety, SB = Sociotechnical blindness, TSE = Technology self-efficacy, ATI = Affinity for technology interaction, PI = Professional identification, AGE = Age, GEN = Gender, OP = Openness, CO = Conscientiousness, EX = Extraversion, AG = Agreeableness, NE = Neuroticism | | | | | | | | | | |

| **Table A10**  *Structural equation modeling results with control variables for the feedback tool* | | | | | | | | | | |
| --- | --- | --- | --- | --- | --- | --- | --- | --- | --- | --- |
|  | β | SE | 95-CI | *p* | H | β | SE | 95-CI | *p* | H |
| LI → UI | **.754** | 0.033 | [.69, .82] | **.000** | H1 |  |  |  |  |  |
| *Effects on LI* | |  |  |  |  | *Effects on UI* | |  |  |  |
| CR | **.145** | 0.076 | [-.00, .30] | **.057** | H2a | .119 | 0.119 | [-.12, .35] | .319 | H3a |
| VR | .016 | 0.067 | [-.11, .15] | .811 | H2b | -.116 | 0.105 | [-.32, .09] | .267 | H3b |
| ER | -.089 | 0.109 | [-.31, .12] | .367 | H2c | **.573** | 0.178 | [.22, .92] | **.001** | H3c |
| LA | -.046 | 0.042 | [-.13, .04] | .273 | H2d | -.008 | 0.066 | [-.14, .12] | .904 | H3d |
| JA | -.086 | 0.077 | [-.24, .06] | .262 | H2e | .085 | 0.120 | [-.15, .32] | .479 | H3e |
| SB | .123 | 0.066 | [-.01, .25] | .062 | H2f | -.077 | 0.103 | [-.28, .13] | .456 | H3f |
| TSE | -.094 | 0.118 | [-.33, .14] | .426 | H2g | -.231 | 0.186 | [-.60, .13] | .215 | H3g |
| ATI | .004 | 0.055 | [-.10, .11] | .940 | H2h | **.182** | 0.087 | [.01, .35] | **.035** | H3h |
| **PI** | **.134** | 0.048 | [.04, .23] | **.006** | RQ5 | -.034 | 0.075 | [-.18, .11] | .648 | RQ6 |
| AGE | -.009 | 0.004 | [-.02, -.00] | **.014** |  | -.001 | 0.006 | [-.01, .01] | .873 |  |
| GEN | -.013 | 0.092 | [-.02, .17] | .884 |  | -.088 | 0.146 | [-.37, .20] | .548 |  |
| OP | .016 | 0.043 | [-.07, .10] | .717 |  | .001 | 0.068 | [-.12, .13] | .984 |  |
| CO | .018 | 0.053 | [-.09, .12] | .730 |  | -.083 | 0.083 | [-.25, .08] | .320 |  |
| EX | -.083 | 0.042 | [-.17, -.00] | **.048** |  | -.011 | 0.066 | [-.14, .12] | .865 |  |
| AG | -.032 | 0.053 | [-.14, .07] | .537 |  | .161 | 0.083 | [-.00, .32] | .051 |  |
| NE | -.009 | 0.043 | [-.10, .08] | .838 |  | .110 | 0.068 | [-.02, .24] | .106 |  |
| *Notes*. LI = Learning intention, UI = Use intention CR = Cognitive readiness, VR = Vision readiness, ER = Ethical readiness, LA = Learning anxiety, JA = Job replacement anxiety, SB = Sociotechnical blindness, TSE = Technology self-efficacy, ATI = Affinity for technology interaction, PI = Professional identification, AGE = Age, GEN = Gender, OP = Openness, CO = Conscientiousness, EX = Extraversion, AG = Agreeableness, NE = Neuroticism | | | | | | | | | | |

| **Table A11**  *Structural equation modeling results with control variables for the practice management tool* | | | | | | | | | | |
| --- | --- | --- | --- | --- | --- | --- | --- | --- | --- | --- |
|  | β | SE | 95-CI | *p* | H | β | SE | 95-CI | *p* | H |
| LI → UI | **.698** | 0.034 | [.62, 76] | **.000** | H1 |  |  |  |  |  |
| *Effects on LI* | |  |  |  |  | *Effects on UI* | |  |  |  |
| CR | -.035 | 0.068 | [-.17, .10] | .607 | H2a | .057 | 0.107 | [-.15, .27] | .592 | H3a |
| VR | .026 | 0.059 | [-.09, .14] | .657 | H2b | -.057 | 0.093 | [-.24, .13] | .539 | H3b |
| ER | .021 | 0.096 | [-.17, .21] | .830 | H2c | **.578** | 0.159 | [.27, .89] | **.000** | H3c |
| LA | -.067 | 0.038 | [-.14, .01] | .073 | H2d | .002 | 0.059 | [-.11, .12] | .977 | H3d |
| JA | -.117 | 0.068 | [-.25, .02] | .088 | H2e | .008 | 0.107 | [-.20, .22] | .938 | H3e |
| SB | **.145** | 0.059 | [.03, .26] | **.014** | H2f | -.022 | 0.092 | [-.20, .16] | .807 | H3f |
| TSE | -.039 | 0.106 | [-.25, .17] | .714 | H2g | **-.394** | 0.169 | [-.72, -.06] | **.020** | H3g |
| ATI | .026 | 0.050 | [-.07, .12] | .598 | H2h | **.191** | 0.078 | [.04, .34] | **.014** | H3h |
| **PI** | .081 | 0.043 | [.00, .17] | .059 | RQ5 | -.015 | 0.068 | [-.15, .12] | .828 | RQ6 |
| AGE | -.013 | 0.005 | [-.02, -.00] | .008 |  | -.013 | 0.005 | [-.02, -.00] | .011 |  |
| GEN | -.371 | 0.121 | [-.61, -.13] | .002 |  | -.374 | 0.130 | [-.63, -.12] | .004 |  |
| OP | .065 | 0.056 | [-.05, .18] | .249 |  | .039 | 0.060 | [-.08, .16] | .522 |  |
| CO | .069 | 0.069 | [-.07, .21] | .315 |  | -.045 | 0.074 | [-.19, .10] | .545 |  |
| EX | .075 | 0.055 | [-.03, .18] | .172 |  | .129 | 0.059 | [-.01, .24] | .029 |  |
| AG | -.045 | 0.069 | [-.18, .09] | .513 |  | .001 | 0.073 | [-.14, .14] | .994 |  |
| NE | .017 | 0.057 | [-.10, .13] | .766 |  | .030 | 0.061 | [-.09, .15] | .624 |  |
| *Notes*. LI = Learning intention, UI = Use intention CR = Cognitive readiness, VR = Vision readiness, ER = Ethical readiness, LA = Learning anxiety, JA = Job replacement anxiety, SB = Sociotechnical blindness, TSE = Technology self-efficacy, ATI = Affinity for technology interaction, PI = Professional identification, AGE = Age, GEN = Gender, OP = Openness, CO = Conscientiousness, EX = Extraversion, AG = Agreeableness, NE = Neuroticism | | | | | | | | | | |
